# The eight pillars of within-host tuberculosis modelling

**DOI:** 10.3389/fimmu.2026.1920524

**Published:** 2026-09-03

**Authors:** Roselyn F. Kaondera-Shava, Christian T. Michael, Denise Kirschner, Ruth Bowness

**Affiliations:** 1 Centre for Mathematical Biology, Department of Mathematical Sciences, University of Bath, Bath, United Kingdom; 2 Department of Microbiology and Immunology, University of Michigan–Michigan Medicine, Ann Arbor, MI, United States

**Keywords:** granuloma, precision medicine, TB coinfection and comorbidity, tuberculosis, within-host mathematical modelling

## Abstract

Tuberculosis (TB) remains a global health challenge, driven by multiscale pathogenesis across molecular, cellular, tissue, and systemic levels. Within-host mathematical modelling provides a powerful way to explore these processes, providing mechanistic insights that are difficult to capture experimentally, including host-pathogen interactions, immune regulation, and treatment efficacy. This review synthesises the evolution of TB models from early ordinary differential equation systems to agent-based and hybrid multiscale approaches, leading to integrated pharmacokinetic/pharmacodynamic and systems pharmacology frameworks. We highlight advances in omics-driven personalisation, virtual clinical trials for regimen optimisation, multiscale and multicompartment extensions, translational impact, evolutionary dynamics, and efforts to improve reproducibility. We also examine modelling strategies for TB coinfections and comorbidities, including HIV and diabetes, which reshape granuloma biology and drug exposure. Unlike previous reviews, we integrate historical and recent within-host tuberculosis modelling advances and present quantitative visual summaries in the form of theme-by-approach heatmaps. In this work, we identify emerging opportunities and current gaps in the landscape of TB models. In addition, we develop a unique workflow for this review that can be adapted for other modelling topics. Our synthesis provides a roadmap towards computational models that may support future precision therapy and TB control strategies.

## 1 Introduction

Tuberculosis (TB) remains one of the most challenging infectious diseases worldwide, causing millions of deaths each year despite the availability of treatment options (1). In 2024 alone, an estimated 10.7 million people developed TB and 1.23 million people died of TB, making it the leading cause of death by single infectious pathogen worldwide (2). The complexity of pulmonary TB lies in the ability of *Mycobacterium tuberculosis* (Mtb) to persist within the host, leading to the formation of multiple *granulomas*. Granulomas are spatially organised immune structures that both contain and harbour Mtb; they are most often found in the lungs and draining lymph nodes of Mtb-infected individuals and exhibit substantial within- and between-host heterogeneity (3–5). Mtb infection and the related immunology determining outcomes are shaped by the anatomical site of infection (6, 7), coinfections (8) and comorbidities (9, 10), and host factors including age (11) immunocompetence (12), immune naivety (13), sex (14), and genetics (15–18). Comorbidities are significant because a large proportion of the global population already harbour latent TB infection (2), creating a substantial reservoir at risk of reactivation. Such reactivation may be triggered by comorbid conditions or coinfections, including HIV, diabetes, undernutrition, or viral infections such as COVID-19, which can weaken immune responses and drive progression from latent infection to active disease (8–10). Thus, TB outcomes are emergent from a tangle of immune mechanisms and bacterial survival strategies interacting across multiple spatial and temporal scales.

Experimental studies have provided invaluable insights into the pathogenesis of TB, but they face significant limitations in capturing processes that occur deep within tissues or unfold over long timescales (19, 20). *In vivo* research on TB is further constrained by ethical considerations (19, 21). The mouse has been the most widely used animal model even though it does not follow the same infection trajectories as humans (22). Two specific mouse models, the C3HeB/FeJ (Kramnik) mouse model and the Ultra Low Dose Model are capable of reproducing granulomas, although disease progression differs from humans (23, 24). Rabbit models have also contributed valuable insights, particularly in reproducing aspects of caseating granuloma formation and latent infection (25). Detailed animal models involve nonhuman primates, as they most closely reproduce human-like granulomas, captures latent TB infection, and support detailed studies of immune responses from early infection through latent and active disease (26). Animal studies must demonstrate a clear scientific justification and comply with strict welfare and regulatory standards, which limit the frequency and duration of such experiments (27, 28). Detailed pharmacokinetic and pharmacodynamic (PK/PD) data are often limited to single timepoint data or to *in vitro* data (29, 30). Human clinical studies face even stronger ethical boundaries: participants cannot be intentionally infected with Mtb, and treatment cannot be delayed or withdrawn (31, 32). As a result, a limited range of TB disease progression and treatment processes can be directly studied *in vivo* (19, 33).

Traditional infectious disease modelling approaches, typically well-mixed compartmental ordinary differential equation (ODE) models, fall short since they assume homogeneous tissues and uniform immune responses (34, 35). These simplified formulations cannot represent TB granuloma heterogeneity, spatial microenvironments, or species-specific immune mechanisms that shape Mtb infection dynamics. Modern detailed within-host modelling helps address these gaps by explicitly simulating granuloma architecture, detailed cellular mechanisms, immune-pathogen interactions, and PK/PD processes that are difficult to capture experimentally (36, 37). These approaches provide two major advantages over earlier models. First, they allow *in silico* testing of more nuanced mechanistic hypotheses, which may help inform treatment regimen optimisation and prediction of treatment outcomes. Second, within-host models help bridge recent discoveries in cellular immune mechanisms with host-scale infection outcomes. These advances highlight the potential contribution of within-host modelling to future personalised TB treatment strategies.

Early TB modelling efforts relied mainly on ODE systems to represent systemic immune responses and bacterial growth, with examples including models by Wigginton and Kirschner (35) and by Marino and Kirschner (38). Although these models provided foundational insights into cytokine regulation and macrophage activation, they relied on well-mixed assumptions, effectively treating immune cells and cytokines as uniformly distributed and lacking the spatial resolution required to capture granuloma-level heterogeneity. This gap motivated the development of agent-based models (ABMs) and hybrid multiscale approaches that integrate molecular signalling, cellular interactions, and tissue architecture (39, 40). More recent advances incorporate PK/PD principles and systems pharmacology, extending existing frameworks for translational applications such as regimen design and virtual clinical trials (41–43).

Previous reviews have made valuable contributions by synthesising key modelling approaches and establishing the foundational landscape of within-host TB research. In 2017 Kirschner et al. (36) consolidated the early ODE, ABM, and hybrid frameworks, providing essential insights into immune regulation and granuloma formation while identifying important challenges in multiscale integration, reproducibility, and pharmacology. Chakraborty et al. (37) provided a concise overview of the main TB modelling approaches, offering brief descriptions of the main modelling families and categorising methods such as ODE-based, agent-based, and hybrid models. As such, their review serves as an accessible entry point for researchers new to the field; however, it focuses primarily on summarising existing frameworks and methods for parameter estimation for ODE-based approaches. A key purpose of this review is to highlight emerging opportunities and promising directions for future work, particularly in areas such as mechanistic modelling of coinfections and comorbidities (e.g. TB-HIV, TB-diabetes, TB-COPD), translational pipelines (tools like GEODE (44), which link *in vitro* data to granuloma-scale simulations and then to virtual patient cohorts) and deeper multiscale integration that connects molecular, cellular, tissue, granuloma, and systemic levels in a consistent and reproducible way.

**Figure 1:**
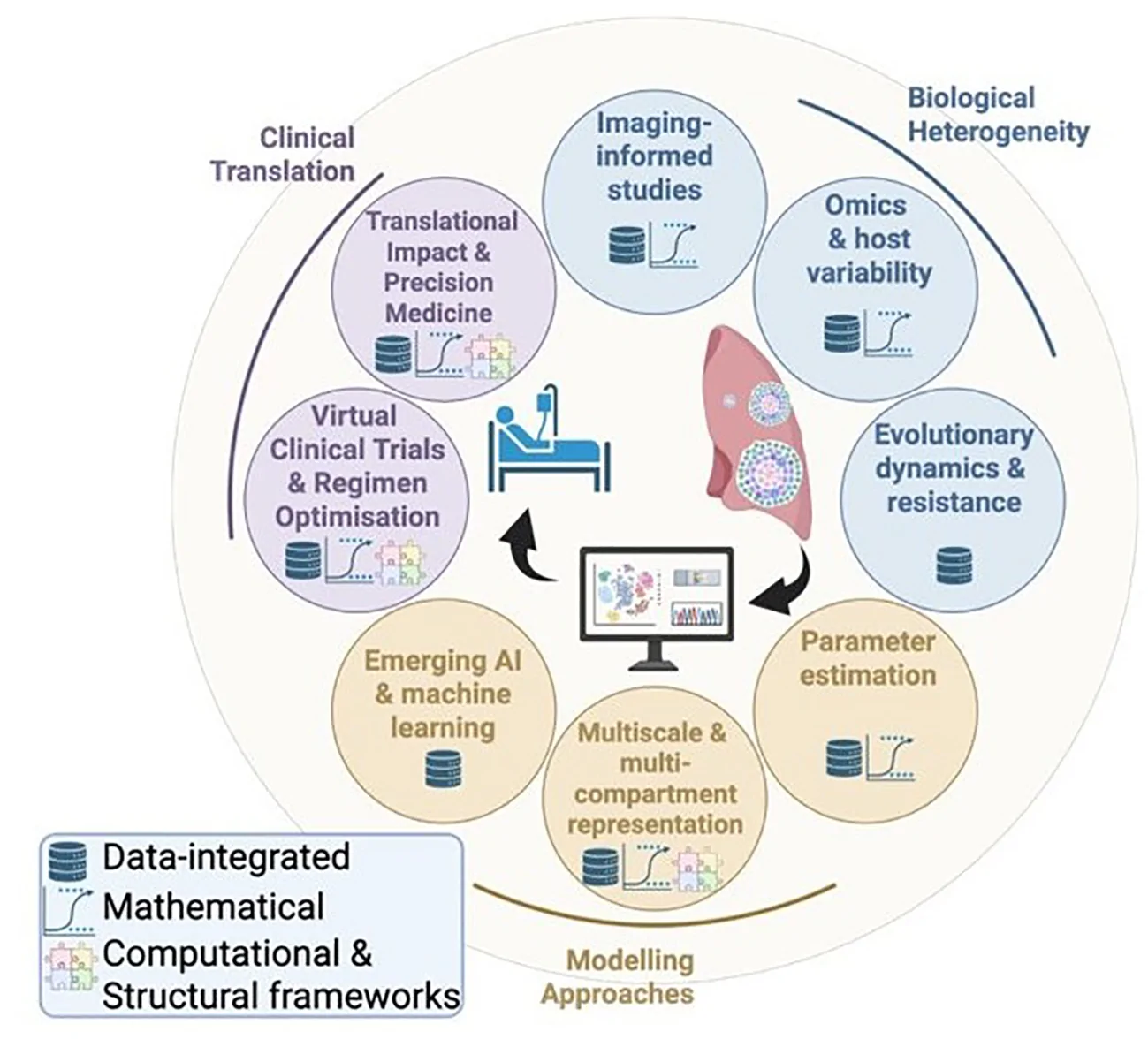
Conceptual overview of post-2017 within-host TB modelling. The framework comprises eight thematic pillars, developed by the authors to organise and understand the main research areas in within-host TB modelling. The Eight Pillars framework emerged from thematic mapping of recurring research themes identified across the reviewed literature. The thematic pillars represent major areas of within-host TB research (i.e. what is being studied), are broadly focused on elucidating biological heterogeneity (blue), developing new methods for modelling TB (yellow), and translating model outcomes toward clinical application (purple). Various computational and mathematical approaches have been used to investigate those themes. Here we group multiple approaches into three families (icons beneath each pillar). Individual studies may contribute to multiple pillars, reflecting the interconnected nature of within-host TB modelling. Detailed descriptions of the approach families and associated modelling techniques are provided in **Table 1**. Created in BioRender (BioRender.com).

Most recent efforts in within-host TB modelling have focused on refining treatment of TB and using novel computational approaches to increase predictive power. We have categorised these papers into eight non-exhaustive and non-mutually-exclusive groups that capture the bulk of recent work (**Figure 1**): integration of omics and host variability, virtual clinical trials and regimen optimisation, multiscale and multicompartment extensions, translational impact and precision medicine, imaging-informed modelling, evolutionary dynamics and resistance, emerging AI/machine-learning approaches, parameter estimation. The small body of research falling outside of these groups largely focused on coinfections including TB-HIV coinfection, TB-diabetes mellitus, TB-COPD and undernutrition.

We structure the review around two distinct time periods, pre-2017 and post-2017, since 2017 marks a significant shift in approaches, tools, and priorities used in within-host TB modelling (36). Prior to this, most models were single-scale with limited pharmacology integration. Post-2017 studies addressed these gaps by introducing omics-driven personalisation, virtual clinical trials, PDE-based transport, and uncertainty quantification workflows (36, 45–47) making 2017 a key pivotal point for structuring this review. We first provide a brief account of the evolution of within-host TB modelling from early ODE frameworks to modern multiscale systems, then emphasise post-2017 advances that address limitations of those early models. We then review post-2017 TB modelling efforts, beginning with those grouped by the eight thematic areas shown in **Figure 1**.

Strikingly, very few mechanistic models have been developed to study TB in the context of coinfections and comorbidities; since the first TB-HIV coinfection model was published in 1999 (34), a small number of mechanistic studies have explored TB-HIV, TB-diabetes, or TB-COPD interactions, making this one of the most underdeveloped areas in within-host TB modelling. This gap is important given the substantial global burden of latent TB infection, with approximately a quarter of the global population (approximately 1.7 billion individuals of the 8.2 billion) harbouring latent infection (48), coupled with the impact of coinfections and comorbidities, which can significantly alter immune responses, drive progression to active disease, and influence treatment outcomes (9, 49, 50). We examine modelling approaches for studying TB with coinfections and comorbidities, including HIV and diabetes, which introduce additional complexity to host immune responses and treatment dynamics (9, 49, 50). These settings highlight the need for more mechanistic and integrative models that can capture interactions and treatment effects across biological scales. We conclude by highlighting open problems and pose a forward-looking roadmap for integrating comorbidity modules and improving reproducibility standards with the potential to inform future personalised treatment strategies.

## 2 Methods

### 2.1 Review rationale and methodological framework

This review provides an overview of the within-host TB modelling literature, identifies major research themes, and highlights key knowledge gaps and future opportunities. The aim is to organise a diverse body of studies into a framework that highlights major research themes, emerging trends, and underexplored areas. This review is a structured narrative and landscape review informed by scoping and evidence-mapping principles. To support transparency and consistency, this review is informed by the JBI Manual for Evidence Synthesis (51), Preferred Reporting Items for Systematic Reviews and Meta Analyses Extension for Scoping Reviews (PRISMA ScR) reporting recommendations (52), Synthesis Without Meta-analysis (SWiM) guidance for narrative synthesis and reporting (53), and Scale for the Assessment of Narrative Review Articles (SANRA) criteria for narrative review quality (54). These frameworks inform study identification, thematic organisation, synthesis, and reporting while retaining the flexibility needed to identify recurring research themes, reveal underexplored areas, and highlight future research opportunities.

To organise and visualise the literature, we use a structured evidence-mapping workflow. Consistent with evidence-mapping approaches, the workflow emphasises breadth of coverage, transparent study selection, thematic mapping, and visual presentation of findings. The resulting thematic classifications and heatmaps represent an author-curated synthesis of the literature and should not be interpreted as an exhaustive description of the field. Rather, they are intended as transparent organisational tools for identifying patterns, concentrations of research activity, and knowledge gaps within the within-host TB modelling landscape.

**Figure 2:**
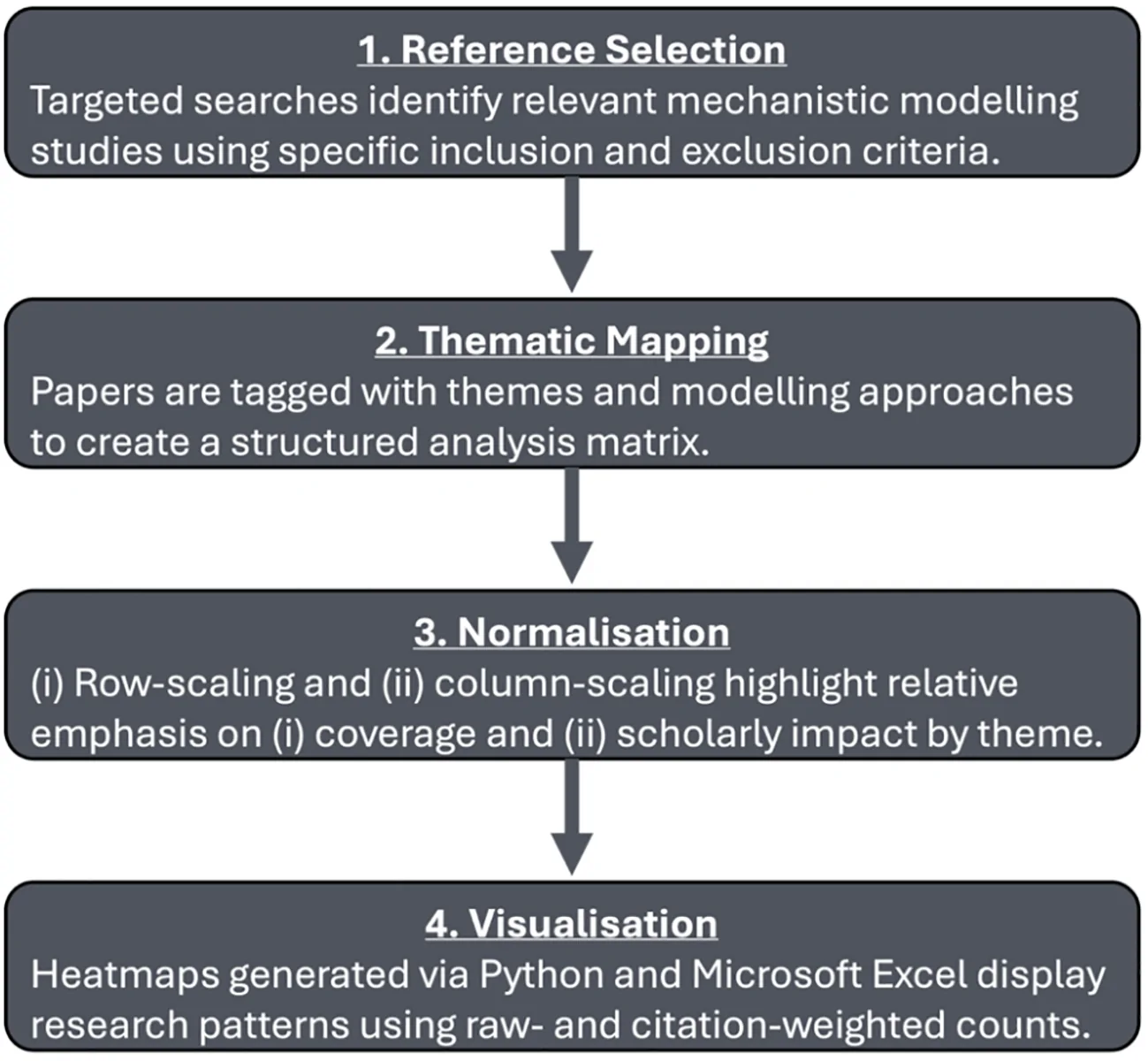
Structured workflow schematic.

Our review framework is divided into four main steps: Reference selection, thematic mapping, normalisation, and visualisation (**Figure 2**). Reference selection describes how relevant literature was identified (Section 2.2). Thematic mapping organises each study into one or more of the eight thematic pillars shown in **Figure 1** (Section 2.3). Normalisation incorporates both raw count and citation-weighted analyses (Section 2.4). Visualisation and reporting describe how these data are aggregated and presented (Section 2.5).

The fit-for-purpose evidence-mapping workflow provides a transparent framework for organising a diverse and highly interconnected literature and for identifying patterns and knowledge gaps. To ensure transparency in the visual synthesis, we align with SWiM guidance by explicitly reporting how studies are grouped (*Themes ⨉ Approaches*), the metrics used (raw count and citation-weighted analyses), the synthesis methods used, and the presentation of findings through heatmaps.

Because many within-host TB modelling studies address multiple research questions, assigning studies to thematic pillars occasionally required informed judgement by the authors. Individual studies may therefore contribute to more than one thematic pillar. The Eight Pillars provide a practical framework for organising the literature, and alternative frameworks could also be developed. Finally, SANRA criteria inform the narrative synthesis by promoting clear aims, transparent reporting of the literature, appropriate use of references, and coherent interpretation of findings.

### 2.2 Reference selection

#### 2.2.1 Sources and search strategy

We conducted initial targeted searches using keywords such as “within-host tuberculosis modelling” and “granuloma agent-based model” in Google Scholar (last accessed: June 2026) to identify relevant studies and capture the current state of within-host TB modelling. To broaden coverage and improve reproducibility, we then extended the search across PubMed, Scopus, and the Web of Science Core Collection, using database appropriate field logic and a shared conceptual structure combining tuberculosis, within-host or granuloma-scale processes, and mathematical or computational modelling approaches.

To ensure coverage of historical frameworks, we seeded the search with the 2017 review by Kirschner et al. (36), which served as a foundational reference for earlier within-host TB modelling. We expanded around this seed using Connected Papers (55) to explore the local citation network and identify influential predecessors and methodological precursors.

For post-2017 advances, we perform searches separately by theme using a fixed core modelling block (52), ensuring that the approaches were kept constant while themes varied for the *Theme ⨉ Approach* mapping. We screened all retrieved papers at the title-abstract level, and those identified as relevant were included in the review. We did not tag review articles to themes or approaches because they synthesise rather than implement modelling frameworks. Rather, they are used to contextualise trends and clarify how modelling themes have evolved. We used Google Scholar in combination with curated databases to improve coverage of interdisciplinary research outputs not consistently indexed in PubMed, Scopus, or Web of Science. We used Google Scholar to obtain citation counts because it covers more types of publication and updates citations faster than curated databases.

#### 2.2.2 Eligibility and screening

To ensure relevant references, we define inclusion criteria for references in this review. We screened titles and abstracts for relevance, followed by full text assessment. We included studies that meet at least one of the following criteria:

1. Introduced or extended a modelling framework relevant to within-host TB, such as ordinary differential equation (ODE) systems, partial differential equation (PDE) systems, agent-based models (ABMs), hybrid ABM-ODE/PDE approaches, or physiologically based pharmacokinetic (PBPK) and quantitative systems pharmacology (QSP) models.
2. Provided calibration and validation, including spatial drug distribution, oxygen and nutrient transport, or virtual cohort sensitivity analyses.
3. Demonstrated data-anchored pharmacology, incorporating PK/PD interactions and regimen optimisation.
4. Presented systematic reviews or methodological guides applicable to within-host TB modelling.

We exclude population-level epidemiological models that focus solely on transmission, as they do not capture within-host processes, along with narrative articles that lack mechanistic modelling or validation.

### 2.3 Thematic mapping

We identified thematic areas by noting recurring patterns in TB-related scientific questions addressed across studies and were then manually refined into a three-category thematic structure: (1) biological TB questions that focus on within-host disease mechanisms (such as granuloma dynamics, host variability, and the evolution of drug resistance), (2) translational TB questions that capture how within-host models are used to inform treatment strategies and clinical decision making (such as virtual clinical trials and precision medicine applications) and (3) non-biological but TB-relevant challenges, such as diagnostics, inference methodologies, and data-driven discovery approaches that shape how within-host TB models are informed, constrained, and validated. Although imaging-based studies (such as PET/CT, histology) and animal models do not constitute computational models themselves, they provide important biological and experimental data that inform and validate TB models.

The Eight Pillars framework emerges from recurring themes and research questions identified across the reviewed literature. Because many studies address multiple research questions, thematic assignment occasionally requires informed judgement, and individual studies may contribute to multiple pillars, and alternative ways of organising the literature are possible.

On the other hand, modelling approaches are used here as an umbrella term for the computational and mathematical methods used to address the thematic areas, including both model frameworks and supporting methodologies such as ODE/PDE models, PBPK/QSP models, ABMs, and surrogate or machine-learning-assisted modelling. This distinction ensures a clear separation between what TB problem(s) is/are being addressed (themes) and how it is tackled methodologically (approaches). Each relevant article was consistently tagged with one or more thematic areas and modelling approaches. These tags provided the structure for aggregating, normalising, and visualising results in Section 4. We perform similar analyses in Section 5, instead tagging articles by pathogen of coinfection and/or comorbidity.

### 2.4 Normalisation

Considering raw- and citation-weighted metrics provides a balanced view of current research coverage and highlight areas for potential future work. We compute raw counts by tallying the number of papers mapped to each *Theme ⨉ Approach* cell. To capture relative emphasis, we applied two normalisation schemes. First, we perform *row normalisation*, where each theme row was scaled so that its values sum to one, highlighting the distribution of approaches within that theme. Second, we perform *column normalisation*, where each approach column is scaled so that its values sum to one. This highlights the contribution of themes to each approach. Cells with zero counts remained zero after normalisation. Examples are provided in **Appendix A**.

To complement raw counts, we computed citation-weighted heatmaps to incorporate scholarly impact. Because citation-weighting can bias toward older studies, we apply section-specific normalisation to reduce distortion from highly cited outliers and maintain fair comparisons within each time frame. We scale the contribution of each article by its Google Scholar citation count using the formula: *w_i_* = *c_i_/c*_max,_ where *c_i_* is the citation count for article *i* and *c*_max_ is the section specific maximum (Section 4 or Section 5). Articles with zero citations contributed a weight of zero. We obtain the weighted values for each *Theme ⨉ Approach* cell by summing weights of all articles assigned to that cell. We apply row and column normalisation as previously described for raw counts. We provide example weight computations in **Appendix Table A5**.

This review’s primary findings are based on the thematic mapping and raw count analyses. The citation-weighted heatmaps are supplementary analyses that provide an additional perspective and help contextualise patterns of scholarly impact across the literature. Citation counts should be interpreted cautiously, as they may be influenced by factors beyond scientific contribution, including publication age, journal visibility, field size, and citation practices.

### 2.5 Visualisation

**Figure 3:**
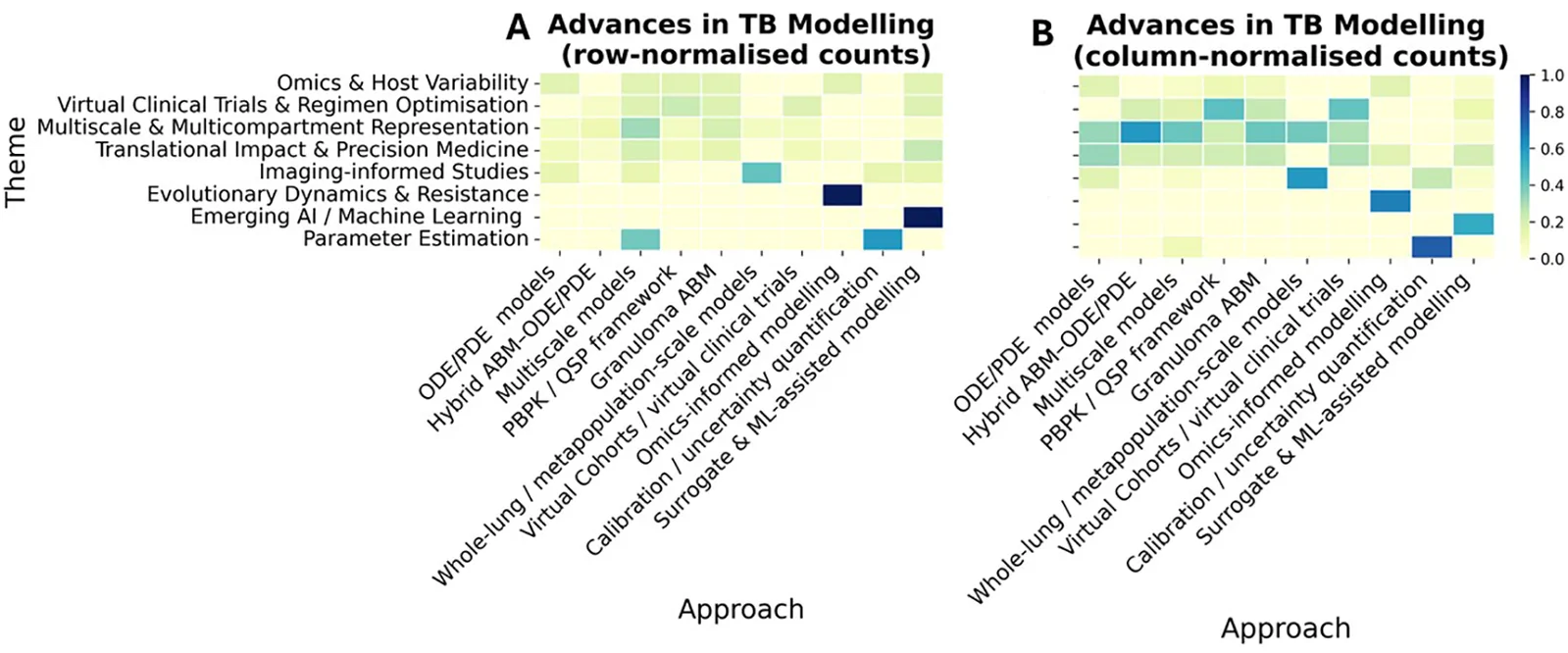
Research coverage of post-2017 modelling studies by theme and approach. Heatmaps normalised by row **(A)** and column **(B)** of modelling approaches across post-2017 TB research themes (Section 4), based on raw counts of selected studies (number of publications). The x-axis represents modelling approaches, and the y-axis represents post-2017 research themes. In **(A)**, each row is normalised to sum to 1, showing the proportion of each modelling approach within a given research theme. In **(B)**, each column is normalised to sum to 1, illustrating how each research theme contributes to a given modelling approach. The intensity of the colour (as indicated by the colour bar) represents the normalised proportion of the raw counts (range 0-1), with lighter colours indicating lower research coverage and darker colours indicating higher research coverage. These heatmaps reflect research coverage across the selected studies. The citation-weighted versions, reflecting the impact of the studies, are presented in **Appendix Figure A1**.

**Figure 4:**
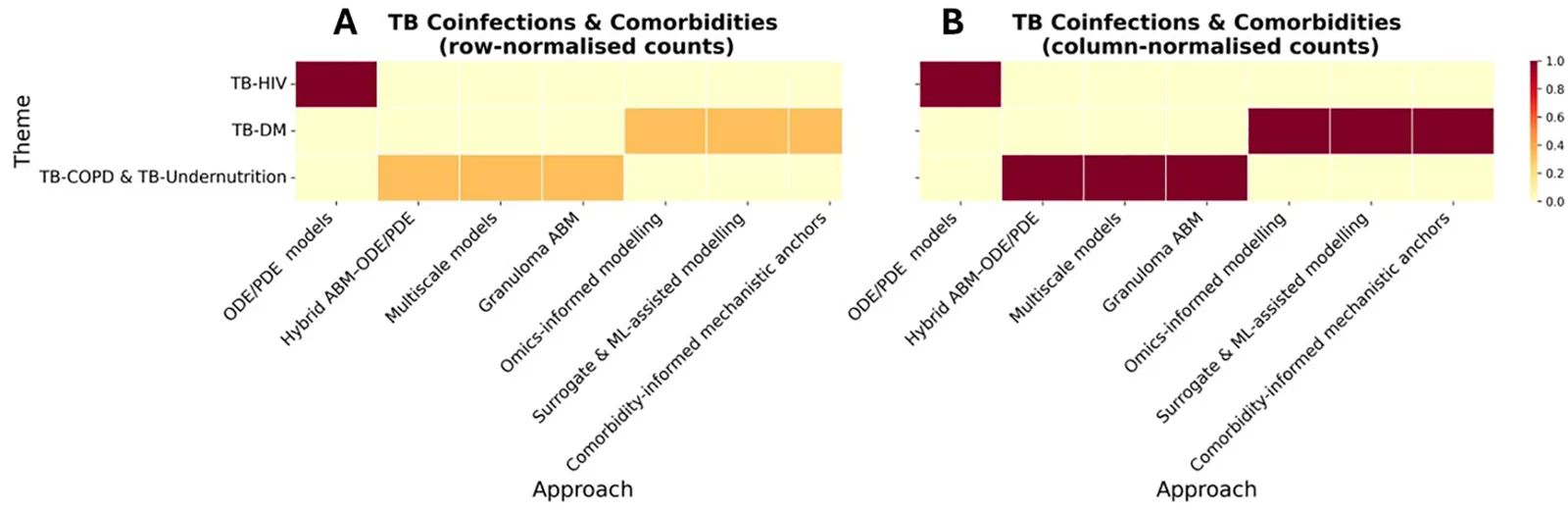
Research coverage of TB modelling studies across coinfections and comorbidities by modelling approach. Heatmaps normalised by row **(A)** and column **(B)** of modelling approaches across TB coinfections and comorbidities (Section 5), based on raw counts of selected studies (number of publications). The x-axis represents the modelling approaches, and the y-axis represents the TB coinfection and comorbidity themes. In **(A)**, each row is normalised to sum to 1, showing the proportion of each modelling approach within a given comorbidity theme. In **(B)**, each column is normalised to sum to 1, illustrating how each comorbidity theme contributes to a given modelling approach. The intensity of the colour (as indicated by the colour bar) represents the normalised proportion of the raw counts (range 0-1), with lighter colours indicating lower research coverage and darker colours indicating higher research coverage. These heatmaps reflect the research coverage across the selected studies. The citation-weighted versions, reflecting the relative influence of the studies, are provided in **Appendix Figure A2**.

We first compile heatmaps in Excel for initial tabulation and then finalised in Python for visualisation. We provide scripts and data tables in **Supplementary Material**. We generated both raw count and citation-weighted analyses (see **Figures 3**, **4**; **Appendix Figures A1, A2**). The heatmaps are illustrative visual summaries of the thematic organisation of the reviewed literature rather than exhaustive representations of the field. They are intended to highlight dominant patterns, expose gaps, and help prioritise promising areas for future work in within-host TB modelling.

### 2.6 Reproducibility measures

To ensure transparency and reproducibility, we document each stage of the workflow, including tools used, information sources, eligibility/selection decisions, and the data-charting fields applied during thematic mapping, in line with scoping review reporting expectations (PRISMA ScR and JBI Scoping Reviews Manual (51, 52)). For quantitative synthesis and visuals, we report the grouping structure (*Theme ⨉ Approach*), the metrics used (raw counts; citation-weighted), the normalisation steps (row/column scaling), the heatmap presentation, and the limitations of these summaries, consistent with the SWiM (53) reporting guideline for non meta-analytic reviews. We provide all scripts, raw data tables, and normalised matrices in the **Supplementary Material** to support full replication.

## 3 Historical context of within-host TB modelling (pre-2017)

### 3.1 Foundational models

Within-host TB modelling has evolved substantially over the past two decades, driven by the need to capture complex host-pathogen interactions and inform therapeutic strategies. Early models, developed in the late 1990s (34) and early 2000s (35), primarily used ODEs to represent systemic immune responses and bacterial dynamics. The foundational work by Wigginton and Kirschner (35) provided critical insights into cytokine regulation and macrophage activation, establishing the importance of cytokines in bacterial containment. However, ODE-based models lack spatial resolution, limiting their ability to represent localised processes involved in granuloma formation.

This gap was addressed by Segovia-Juarez et al. (39) in 2004, who pioneered the first ABM for TB granulomas, *GranSim*. While there had been a previous with-host ABM of shock developed by An in 2001 (56), spatial dynamics were not a key feature of that work. The granuloma ABM was used to simulate local immune-cell interactions and spatial heterogeneity, revealing that the granulomas are dynamic structures whose stability depends on immune cell recruitment and cytokine balance. Building on this innovation, hybrid multiscale models emerged, integrating ABMs with ODEs and partial differential equations (PDEs) to link molecular signalling with tissue-level outcomes. Notably, Fallahi-Sichani et al. (57) demonstrated that tumour necrosis factor *α* (TNF-*α*) signalling and its regulatory dynamics govern granuloma stability.

From 2015 onward, the field began to incorporate different resolutions of pharmacology into these frameworks. Systems pharmacology models combined the principles of PK/PD with multiscale modelling to explore drug penetration barriers and regimen efficacy (41–43). These studies captured how the structure of the granuloma, particularly the necrotic, hypoxic centre (caseum) slows drug movement and makes antibiotics less effective, as observed experimentally (29, 58, 59). They also introduced optimisation algorithms for regimen design, marking a shift toward potential precision medicine. Kirschner et al. (36) provided a landmark synthesis of these developments, framed their biological insights and translational potential.

### 3.2 Advantages and disadvantages of early TB models

Prior to 2017, researchers developed the foundational set of modelling approaches that the TB modelling still relies on today. Those original ODEs, ABMs, and hybrid multiscale models remain foundational within-host TB modelling frameworks.

#### 3.2.1 Advantages of foundational models

Classical ODE models provided fast and mathematically tractable ways to study immune regulation and cytokine dynamics. They made it feasible to explore systemic-level behaviour. For example, the foundational systemic model by Wigginton and Kirschner (35) clarified the roles of TNF-*α* and IFN-*γ* in bacterial containment. ABMs added spatial detail, showing how granulomas form from local cell-cell interactions and emergent behaviour, as in Segovia-Juarez et al. (39), which pioneered molecule-to-granuloma-level simulations. Hybrid multiscale models (ABM coupled to ODE/PDE) combined cellular, molecular, and tissue-scale components, enabling much more realistic representations of granuloma structure and signalling networks; Cilfone et al. (60), for instance, showed how the balance of TNF-*α* and IL-10 governs granuloma stability, an insight that required both cellular interactions and cytokine kinetics. Early systems pharmacology studies provided quantitative insight into granuloma drug exposure and treatment response. Pienaar et al. (41) (2015) used in silico experiments to evaluate antibiotic regimens, Pienaar et al. (42) (2016) linked metabolic/gene perturbations to sterilisation predictions, and Pienaar et al. (43) (2017) compared fluoroquinolones using a multiscale systems pharmacology approach.

#### 3.2.2 Disadvantages of foundational models

Pre 2017 approaches faced important structural (model form) and practical (data/calibration) limitations that constrained inference and motivated later advances. Classical ODE models assumed well mixed interactions and therefore could not represent granuloma-scale spatial heterogeneity evident in host TB, as they were formulated at the systemic scale and treated immune and bacterial dynamics as population averages (35). Early ABMs captured spatial heterogeneity but were parameter-rich and computationally intensive, complicating parameter estimation, uncertainty quantification, and reproducibility, as seen in early granuloma ABMs (39). Although hybrid ABM-ODE/PDE models (60) increase biological realism by coupling cell-level interactions with molecular signalling and spatial diffusion, they also increase model dimensionality and introduce calibration and validation issues, as feedbacks between cellular-, molecular-, and tissue-scale processes can lead to numerically unstable behaviour and high computational cost. Early PK/PD and systems-pharmacology models began to address drug exposure within granulomas, but their predictive power was limited by the scarcity of granuloma specific human PK/PD data and by incomplete representations of drug, oxygen, and nutrient transport. These constraints were acknowledged in the Pienaar et al., 2015–2017 studies (41–43), which highlighted that lack of granuloma-specific human PK/PD data and incomplete transport representations limited the ability to make confident predictions about antibiotic penetration and regimen performance across heterogeneous granuloma environments.

## 4 Contemporary advances in within-host TB modelling

Since 2017, within-host TB modelling has accelerated, propelled by richer datasets (imaging and omics), advanced computational strategies, and calibration methods for complex models (45, 61, 62). These innovations have expanded the field from granuloma-scale detail to systemic integration and translational pipelines (44, 46). Post-2017 within-host TB modelling substantially broadened scope and realism by integrating new data sources, richer spatial and anatomical detail, and more advanced computational methods. These included omics-informed PBPK/QSP models, virtual clinical trials, PDE-based transport, whole-lung modelling, imaging-guided calibration, and AI/ML based optimisation, all of which improved mechanistic understanding and increased the potential for clinically relevant predictions compared with pre-2017 approaches.

To structure this review, we group post-2017 within-host TB modelling papers into eight non-mutually-exclusive, non-exhaustive themes (**Figure 1**) that form the basis of the *Theme ⨉ Approach* mapping and capture the major directions of recent advances (Sections 4.1-4.8). Because within-host TB studies often address multiple biological questions using more than one modelling framework, individual studies can map multiple themes and approaches. These themes are: (1) integration of omics and host variability (45, 63); (2) virtual clinical trials and regimen optimisation (46, 64, 65); (3) multiscale and multicompartment representation (62, 66, 67), contextualised by recent advances in modular multiscale TB systems (47); (4) translational impact and precision medicine (44, 45); (5) imaging-informed studies (61, 68); (6) evolutionary dynamics and resistance (69, 70); (7) emerging AI/machine-learning (44, 71) and (8) parameter estimation (72, 73).

While the themes in **Figure 1** describe the research focus of paper, we also examine eleven *approaches* used to study those themes (**Table 1**). In many cases, theme and approach overlap, as much recent modelling work has focused on exploring capabilities of a given approach. We further grouped those approaches into four families based on the distinct functional roles they play in within-host TB modelling workflows and the natural clustering observed during mapping.

**Table 1:** Contemporary approaches to within-host TB modelling.

Family of approaches	Approach	References
*Mathematical formulations*	ODE/PDE models	(34, 62)
	Hybrid ABM-ODE/PDE	(67, 74)
	Multiscale models	(46, 66)
	PBPK/QSP frameworks	(45, 65)
*Computational and structural frameworks*	Granuloma-based ABM	(44, 67, 75)
	Whole-lung or metapopulation-scale models	(66, 75)
	Virtual cohorts or virtual clinical trials	(46)
*Data-integrated calibration and inference*	Omics-informed modelling	(69, 76)
	Calibration/UQ	(72, 73)
	Surrogate and ML-assisted	(44, 77)
*Mechanistic biological subfields*	Comorbidity-informed mechanistic anchors	(78)

These categories are non-mutually-exclusive and non-exhaustive.

The primary findings of the review are based on the thematic mapping of studies and the resulting raw count heatmaps (**Figure 3**). Citation-weighted heatmaps (**Figure A1**) are presented as supplementary exploratory analyses that provide additional context regarding patterns of scholarly impact. The raw count heatmaps distinguish three broad regions within the literature: mature areas (high coverage), emerging areas, and potential gaps. While these patterns are interpreted within the Eight Pillars framework, they highlight where the field is well-developed, where innovation is accelerating, and where opportunities remain.

Established approaches such as ODE/PDE and PBPK/QSP are used across many themes, indicating that they are used across many different types of within-host TB studies. In contrast, Emerging AI/Machine Learning, Parameter Estimation, and Evolutionary Dynamics & Resistance remain dominated by Surrogate & ML assisted Modelling, Calibration and Uncertainty Quantification, and Omics informed Modelling, respectively, with limited application across the eight themes. Notably, the Evolutionary Dynamics & Resistance theme has been addressed exclusively by Omics informed Models.

Panel A of **Figure 3** shows the modelling approaches most associated with each thematic area. Supplementary citation-weighted analyses are provided in **Figure A1**. In the row-normalised, raw count heatmap (**Figure 3A**), several themes exhibit concentrated associations with specific approaches, reflecting differences in methodological maturity and scope. Parameter estimation and Emerging AI/machine learning display strong alignments with Calibration/UQ and Surrogate & ML-assisted modelling, respectively, indicating that these themes remain largely method-centric. As of now, the primary scientific contributions of these models are methodological, focusing on the development, integration, and refinement of modelling frameworks. This clustering along the diagonal suggests a siloing effect, where the approaches are often applied within their own thematic areas, with limited cross-theme integration. Such a pattern may reflect how emerging approaches influence within-host TB modelling: initially establishing concepts and standards and later becoming more widely adopted as modelling approaches. A distinct pattern is observed for Imaging-informed Studies, which are distributed across other approaches. This reflects the role of imaging data (e.g. PET/CT and histopathology) in informing, constraining, calibrating, and validating multiple types of within-host TB models. In parallel, Virtual clinical trials & regimen optimisation and Translational impact & precision medicine apply multiple approaches (such as PBPK/QSP, ODE/PDE, multiscale), consistent with their application-driven focus.

As a supplementary exploratory analysis, the row-normalised citation-weighted heatmap (**Figure A1A**) provides an indication of how scholarly impact is distributed across themes and approach families. Calibration-focused and AI-driven studies appear concentrated within the Parameter Estimation and Emerging AI/Machine Learning thematic areas, respectively. These patterns largely reinforce patterns already seen in the primary raw count heatmaps.

Panel B of **Figure 3** shows the themes driving the use of each modelling approach. Supplementary citation-weighted analyses are shown in **Figure A1**. The column-normalised, raw count heatmap (**Figure 3B**) shows that the ODE/PDE and PBPK/QSP approaches are used to study multiple themes, indicating that these approaches have matured into a standard reusable modelling infrastructure for within-host TB research. In contrast, studies using Calibration/UQ approach tends to be concentrated within a narrower set of themes, reflecting its current use primarily within parameter estimation contexts. The column-normalised citation-weighted heatmap (**Figure A1B**) suggests that scholarly impact associated with Calibration/UQ, Surrogate & ML-assisted modelling, and Omics-informed modelling approaches remains concentrated within method-focused thematic areas. These citation-weighted results provide additional context on scholarly impact but are not interpreted as the primary findings of the review.

The field has progressed since 2017 in each of the eight thematic areas, due to increased computational speed and methodological advancements in integrating spatiotemporal heterogeneity and large datasets into models. Omics-integrated PBPK/QSP pipelines link drug-induced transcriptomic signatures to physiologically based pharmacokinetic and systems pharmacology models (45). By converting gene-expression responses under drug pressure into quantitative effects on drug handling and immune pathways, these pipelines propagate molecular-level variation through PK/PD layers. This provides a mechanistic interpretation of drug interactions and may support future personalised predictions of treatment outcomes by accounting for host-specific molecular variability. Virtual cohorts and virtual clinical trials provide a platform for systematic exploration across heterogeneous hosts and granulomas, as implemented in *HostSim* (46), while multiscale PDE based transport explains how oxygen, nutrients, and drugs form gradients that shape granuloma outcomes (62). Anatomical scaling is achieved with whole lung/metapopulation models that predict dissemination and regional granuloma patterns (66, 75). Evolutionary and genomics-informed resistance modelling provides insights into how uneven drug exposure within granulomas may drive the emergence and selection of resistant subpopulations (69, 79). Translational pipelines and surrogate based/ML optimisation accelerate regimen ranking and *in vitro* to *in vivo* translation (44, 80). Standardised calibration and UQ workflows (e.g. CaliPro) strengthen identifiability and reproducibility in multiscale models (47), while PET/CT imaging and human histopathology have informed model calibration, these data help anchor models to granuloma structure and activity (61, 68), collectively delivering more mechanistically informed and potentially clinically relevant predictions than were possible pre-2017.

Despite these advances, post 2017 modelling frameworks also introduce significant structural and practical challenges. Multiscale ABM-PDE-PBPK/QSP models are computationally heavy and difficult to calibrate, limiting the ability to run large virtual cohorts and reliably estimate transport-physics parameters (46, 62). Granuloma-specific human PK/PD measurements remain sparse, reducing confidence in drug exposure predictions; this limitation has been noted across translational pipelines, from the foundational systems pharmacology studies (such as Pienaar 2015-2017 (41–43)) to more recent integrated frameworks (44, 46, 62). The mapping of imaging/histology to specific biological processes lacks standardisation, making it challenging to parameterise and validate models even as methods like PET/CT and histology are being increasingly used (47, 61, 62). AI/ML surrogates and optimisation speed up exploration but they need careful validation as they lack the underlying mechanistic biology of the full multiscale models (44, 80). Accurate parameterization remains a central concern in these high dimensional multiscale systems, motivating dedicated calibration workflows (47, 81).

### 4.1 Integration of omics and host variability

Post-2017 advances in biological knowledge and related modelling techniques have increasingly addressed how molecular-scale processes and anatomical variability shape treatment outcomes. Omics data with host-specific physiological differences have provided molecular insights into drug response and immune signalling (37, 45), while host variability captures granuloma architecture and systemic factors that influence antibiotic regimen performance (65). Cicchese et al. (45) demonstrated how differences between granulomas influence treatment response through a multiscale pipeline linking drug transcriptomics to physiologically based pharmacokinetic and quantitative systems pharmacology models (PBPK/QSP), illustrating how molecular responses under drug pressure inform systemic pharmacology and shape predictions of *in vivo* drug-drug interactions. These efforts demonstrate that the integration of omics within PBPK/QSP and granuloma-scale ABMs enhances individualised predictions and highlights the translational potential of TB modelling for precision therapy.

Multi-omics studies provide a biological foundation for incorporating omics informed heterogeneity into mechanistic within-host models. For example, Cox et al. (63) experimentally profiled multiple omics datasets (genomic, transcriptomic, epigenetic and chromatin) features from highly exposed TB contacts, and used multi-omic latent factor integration to identify the key biological pathways associated with Mtb resistance that were not detectable in single-omic analyses. Building on this, the review by Otchere et al. (82) showed that transcriptomic, proteomic, and metabolomic datasets can reveal host-pathogen interaction states that shape immune responses during TB infection. Similarly, in their review of host-derived biomarkers, Cui et al. (83) synthesised how multi-omic signatures reflect differences between individuals in immune response and disease progression. However, standardised workflows for the integration of datasets into mechanistic TB models remains limited, complicated by sparse granuloma-specific PK/PD measurements demonstrated experimentally (29), heterogeneous data types (63), and the computational cost of multiscale simulations (46). Although omics studies provide valuable biological insights, converting these data into mechanistic model parameters that can be reliably estimated and validated remains a challenge.

### 4.2 Virtual clinical trials and regimen optimisation

Virtual clinical trials have emerged as a transformative approach for evaluating TB treatment strategies under diverse host and granuloma conditions without the cost and risk of real-world trials (46, 65). Michael et al. (46) developed the Multiscale Interventional Design (MID) framework to construct virtual patient cohorts and quantify the impact of interventions while accounting for both granuloma and host heterogeneity, identifying host factors such as immune strength and bacterial burden that strongly influence treatment outcomes. Building on this framework, *HostSim* was subsequently used to generate virtual patient cohorts and examine how regimen rankings change under different host conditions (64). Budak et al. (65) used granuloma-scale simulations to screen and rank TB drug combinations, providing an efficient in silico platform for regimen optimisation. Budak et al. (44) introduced GEODE, an in silico decision-support tool that links systematic *in vitro* PK/PD measurements to granuloma-scale simulations, providing a framework for rapid comparison and optimisation of antibiotic combinations within virtual granulomas.

Treatment outcomes depend not only on drugs but also on vaccination-shaped immune responses, suggesting that vaccination status could be incorporated into future virtual clinical trial frameworks when evaluating regimen efficacy. Emerging AI/ML-driven optimisation methods (80) may accelerate virtual trial workflows and inform more personalised treatment strategies. However, harmonised efficacy metrics, robust uncertainty quantification, and real-world validation remain lacking (46, 64). Consequently, virtual clinical trials are not yet routinely used to guide TB treatment in clinical practice.

### 4.3 Multiscale and multicompartment representation

Whole-host TB disease progression is often dependent upon the intra-host heterogeneity of granuloma-scale outcomes (5). At the same time, infection in one granuloma can impact the adaptive immune response to other granulomas within one host (13, 84). Further, granuloma development can be impacted by extremely fine-grain mechanisms and is emergent from a plethora of individual immune components that have each been studied individually (85). While latent granulomas can be reactivated by TNF-*α* inhibitors, the molecular mechanism of that inhibition can result in different rates of reactivation (86, 87). Molecular dynamics of antibiotic binding to caseum can impact pharmacokinetic studies in ways that are challenging to study (29).

To address these challenges, multiscale and multicompartment approaches allow for spatial and systemic integration of multiple mechanisms and datasets for accurate prediction of TB outcomes. Recent work has introduced modular frameworks that integrate granuloma-scale ABMs (e.g. *GranSim*) with PBPK/QSP and host-level simulators such as *HostSim*, creating flexible platforms for cross-scale analysis (37, 47). Michael et al. (46) developed a general methodology (using TB antibiotic treatment as a test case) for determining impacts of interventions at each scale within a multiscale model. Budak et al. (65) used a multiscale systems pharmacology model combining *GranSim* with PK/PD dynamics to capture how heterogeneous granuloma environments affect regimen outcomes.

With recent developments in our ability to simulate and analyse models at the molecular, cellular, granuloma, organ, and whole-host scales has improved predictions of TB progression and treatment response (46, 62, 66, 75). Datta et al. (62) developed PDE-based transport models for oxygen, nutrients, and drugs, showing that the hypoxia and caseum-driven diffusion barriers limit drug penetration and contribute to treatment failure. Pitcher et al. (66) utilised a metapopulation whole-lung model to reproduce the location of apical granuloma and regional heterogeneity. Wessler et al. (75) calibrated MultiGran (a whole-lung hybrid ODE/ABM) to non-human primate data, tracking dissemination and immune control in multiple granulomas. *HostSim* extended this approach by coupling immune-Mtb granuloma dynamics with lymph node and blood compartments to capture the indirect interaction between multiple lung granulomas and concomitant immunity to subsequent infections (13, 88). *HostSim* was then later expanded with PK/PD to study whole-host drug regimen efficacy while capturing drug-drug and immune-drug interactions at the granuloma scale (64). Bowness et al. (67) introduced a hybrid multiscale cellular automaton, highlighting how the state of mycobacteria and their spatial location can alter the efficacy of antibiotic treatment. Marino et al. (89) developed a 3D extension of the *GranSim* hybrid granuloma model and showed that key mechanisms shaping bacterial burden are consistent between 2D and 3D simulations, clarifying how spatial scale influences granuloma behaviour.

At the molecular scale, *GranSim* was later enhanced to represent caseum binding and drug penetration into caseum, as identified experimentally by Sarathy et al. (29), while Cicchese et al. (45) further extended multiscale modelling by incorporating transcriptomic ML outputs into granuloma-scale PK/PD simulations. Oxygen plays a key role in driving both environment- and bacterial-level behaviours within granulomas. Sershen et al. (90) incorporated oxygen dynamics within granulomas, demonstrating that variability in the local environment influences bacterial behaviour and treatment response. A related modelling study by May and Sershen (91) on oxygen availability during latency further highlights how oxygen gradients shape bacterial states and treatment outcomes.

Building and analysing complex multiscale models remains computationally challenging. As model complexity increases, decisions should be made regarding the level of biological detail required to address specific research questions. A process dubbed *Tuneable Resolution*, introduced in 2014, allows models to be built at multiple biological resolutions by adding or coarse-graining detail as needed. This was demonstrated through a whole-granuloma simulation incorporating detailed TNF-*α* dynamics (92).

Special care is taken when modelling multiple scales, as temporal timescales often differ as well; diffusion may happen on the order of seconds while cell motion happens on the order of hours (47, 60). Moreover, two-way coupling between granuloma and systemic dynamics remains fragile and can be computationally expensive (46, 62). Validation of predictions across different biological scales remains limited due to the scarcity of data across scales. Several important immunological processes, including macrophage functional heterogeneity, neutrophil biology, tissue-resident immunity, immunometabolism, granuloma vascularisation, immune exhaustion, and host genetic diversity, remain incompletely represented in current within-host TB models. Improved representation of these mechanisms may strengthen understanding of disease progression, host variability, and treatment response.

### 4.4 Translational impact and precision medicine

Multiscale, pharmacology-integrated models have the potential to inform future clinical decision-making and regimen personalisation. *Digital twins* have been proposed as a personalised modelling approach in which models are calibrated to data from a specific patient (93, 94). This has been explored before in the context of other diseases, such as diabetes (95). However, predicting the trajectory of granuloma formation requires patient data that are not easily samples from blood, and heterogeneity and sparsity of patient-specific TB data make calibration of TB digital twins challenging.

Despite these challenges, multiple efforts have been made to create translational models to study individual aspects of TB treatment in humans. Using a multiscale PK/PD framework, Cicchese et al. (45) showed that synergy against non-replicating bacterial populations was associated with improved regimen performance. Budak et al. (65) provided evidence of translational potential by showing that the ranking of simulated regimens correlated with new marmoset data, suggesting that granuloma-scale modelling may help prioritise TB treatment regimens. Zheng et al. (77) developed a machine-learning virtual screening pipeline to identify two promising repurposed TB drug candidates, demonstrating translational potential for accelerating TB drug discovery. Through sensitivity analyses, Michael et al. (46) showed that immune strength and initial bacterial burden are key predictors of intervention success, underscoring the value of their framework’s relevance for precision-medicine applications. Krupinsky et al. (84) developed a multiscale lymph-node model calibrated to non-human primate data, providing translational insight into how LN-driven mechanisms influence pulmonary infection control. Fonseca et al. (96) generated transcriptomic profiles from human TB lesions and showed that immune and metabolic modules correlate with disease severity and treatment response, providing omics-derived anchors that have the potential to inform precision-medicine stratification. Vaccination status is an additional source of differences between individuals in immune responses, disease progression, and treatment response, highlighting its importance in precision medicine approaches for TB.

Experimental studies by Sarathy et al. (29) showed that caseum binding is a key barrier to drug penetration into necrotic granuloma cores. Building on this foundation, Datta et al. (62) reproduced spatial drug-penetration patterns and evaluated granuloma-targeted approaches such as altering interstitial hydraulic conductivity, providing a framework for exploring granuloma-targeted therapeutic strategies. GEODE (44) further demonstrates how PK/PD-informed modelling frameworks may support translational evaluation of treatment regimens. Budak et al. (80) integrated PBPK/QSP modelling with machine-learning based surrogate optimisation to systematically prioritise TB treatment regimens, identifying alternatives with improved predicted efficacy relative to standard therapy. Using calibrated PBPK/QSP framework (74), they further showed that moxifloxacin-containing regimens exhibit distinct efficacy profiles compared with standard therapy due to non-linear drug interactions and granuloma-level heterogeneity. As a methodological meta-commentary, Michael et al. (64) showed that regimen rankings depend on experimental design choices, including spatial resolution, detection thresholds, and initial bacterial burden, highlighting their influence on reported treatment efficacy and the need for harmonised efficacy metrics across scales. Despite translational pipelines like GEODE, granuloma-targeted delivery strategies and real-world validation remain underdeveloped (44, 62, 64). Routine clinical implementation therefore remains limited, and most within-host TB models remain research tools.

### 4.5 Imaging-informed studies

Imaging provides a bridge between experimental observation and computational modelling by revealing the spatial organisation, heterogeneity, and progression of Mtb infection at granuloma and tissue scales. Although imaging and laboratory techniques such as PET/CT and histology are not computational models themselves, their application across both animal and human studies generates quantitative datasets that inform, constrain, and validate within-host TB models.

Lin et al. (68) demonstrated the value of the PET/CT measurement of metabolic activity in visualising granuloma structure and metabolic activity in non-human primates, generating granuloma-level metrics that inform agent-based and multiscale models. Several imaging-driven studies have further expanded the empirical foundations for imaging informed modelling: White et al. (97) demonstrated the value of FDG PET/CT in macaques for quantifying lesion size, inflammatory activity and lymph node involvement, providing imaging metrics that disease progression, reactivation risk, and antibiotic efficacy. Martin et al. (98) used genome-encoded barcodes together with serial PET/CT to track the fate of individual Mtb lineages in macaques. They showed that while multiple bacterial lineages establish infection, dissemination from primary granulomas is strongly restricted, yielding a high-resolution lesional map that informs modelling of heterogeneous granuloma fates. Maiello et al. (99) used longitudinal PET/CT to characterise relapse under SIV induced reactivation, showing that relapse originates from residual bacterial reservoirs in both lung granulomas and thoracic lymph node lesions, and demonstrating that imaging alone cannot reliably predict relapse. Extending this, Sawyer et al. (61) applied spatial mapping and histopathology to human TB, revealing granuloma diversity and superstructure, which provide insights into granuloma connectivity and progression. Imaging datasets have been incorporated into computational frameworks such as *GranSim*, improving spatial realism in virtual clinical trials and regimen optimization (74). Krupinsky et al. (84) showed that uncertainty and sensitivity analysis of PET/CT data can link lymph-node imaging signals to impaired immune function, thereby providing constraints for mechanistic models. Building on imaging-derived structural data, Datta et al. (62) utilised transport-physics models to mechanistically explain how hypoxia and caseous necrosis restrict drug diffusion within granulomas. Shilpa and Ayeesha Banu (71) showed how CXR-based AI can generate robust, scalable imaging-derived phenotypes that inform within-host TB modelling by enabling disease detection, stratification, and validation against clinically observable imaging signals. However, mappings from PET/CT and histopathology to model parameters remain non-standardised, limiting comparability across studies (61, 62).

### 4.6 Evolutionary dynamics and resistance

Linking variability between granulomas with genomic selection pressures may improve predictions of resistance emergence under variable drug exposure. Castro et al. (79) synthesised evidence that hetero-resistance (coexisting susceptible and resistant subpopulations), phenotypic tolerance (drug surviving, slow/non replicating phenotypes), and microenvironment-mediated selection (when a granuloma environment selects bacterial survival) drive treatment failure and relapse. Morales-Arce et al. (69) provided a population-genomic perspective, showing that within-host Mtb diversity is limited by selective sweeps, purifying selection and progeny skew, and highlighted mutation rates and selection coefficients as critical parameters for calibrating within-host models.

New empirical and genomic studies are generating data that can be used for parameterisation, model fitting, and validation of within-host TB models: Xu et al. (70) performed serial whole-genome sequencing over more than three years in patients with long-standing extensively drug-resistant tuberculosis (XDR-TB), revealing emergence of new resistant subpopulations, clonal competition and turnover, and eventual dominance of a single lineage during ineffective therapy. These longitudinal patterns provide empirical selection dynamics and competition trajectories that can inform strain-explicit within-host models. Worakitchanon et al. (100) analysed clinical Mtb genomes and identified significant resistance-associated variation, thereby expanding the mutational target space that resistance-evolution models should represent. Ismail et al. (76) showed that standard phenotypic testing can detect small bedaquiline-resistant groups down to about 1% when intermediate results are kept, defining realistic initial resistant-subpopulation frequencies and observation thresholds for within-host models and virtual trial outputs. Despite advances in modelling hetero-resistance, integration of genomic selection pressures into multiscale frameworks remains incomplete (69, 79).

### 4.7 Emerging AI/machine-learning

Emerging AI/machine-learning (ML) approaches are increasingly integrated into TB modelling workflows primarily as data-driven tools that complement and inform mechanistic within-host modelling to overcome computational and calibration challenges. Budak et al. (80) introduced a machine-learning-based optimisation framework that efficiently explores regimen combinations using the simulation outputs on granuloma-scale. Similarly, the GEODE pipeline (44) incorporates surrogate modelling to reduce computational cost while ranking regimens, illustrating how hybrid AI-mechanistic approaches operationalise rapid regimen prioritisation rather than relying solely on exhaustive simulations.

AI and ML approaches are being applied to genomic and biomarker datasets, offering new ways to predict drug resistance, drug efficacy and TB diagnostic outcomes that complement mechanistic within-host models. Sharma et al. (101) reviewed ML applications for the prediction of drug resistance in TB, outlining how the growing burden of drug-resistant TB and the slow turnaround of traditional culture-based DST have motivated the use of supervised learning in whole genome sequencing data to detect resistance earlier and more efficiently. Their analysis highlights the potential of ML enabled resistance profiling to address limitations associated with curated mutation databases and improve the identification of drug-resistant TB, particularly in settings where rapid diagnostics are needed.

In TB drug discovery and treatment optimisation, ML frameworks such as INDIGO-MTB (102), developed further in subsequent methodological work (103), together with Larkins-Ford et al. (30, 104) approaches for predicting drug interactions and identifying effective drug combinations, have shown strong potential for identifying effective drug combinations. Sambarey et al. (105) applied ML to predict personalised TB treatment responses, highlighting the potential of data-driven approaches for individualised therapy design. Additionally, Zheng et al. (77) developed a multi-model ML/deep learning virtual screening workflow that successfully identified and experimentally validated two repurposed compounds (aldoxorubicin and quarfloxin) that were shown to work against standard and drug-resistant Mtb. Recently, Whiteley et al. (106) have trained random-forest models to forecast the efficacy of various multi-antibiotic regimens using a synthesis of human-like *in vitro* data and data from marmosets.

At the systems level, ML approaches integrate multiple biological processes (such as immune responses, bacterial dynamics, and metabolism) with diverse data types (including transcriptomic, genomic, and clinical datasets) to understand how they interact and affect treatment outcomes (44, 45, 107, 108). In a recent study, Li et al. (109) used multimodal ML approaches that integrate clinical phenotypes with genomic and molecular data to improve the prediction of individual treatment responses, highlighting the growing role of ML towards precision medicine for TB.

In diagnostics and biomarker discovery, ML improves the diagnosis of TB by using immune-system data to more accurately distinguish between latent and active TB. Reviews by Li et al. (110) and Balakrishnan et al. (111) showed that ML-based biomarker panels can achieve high diagnostic precision and may contribute to the development of faster, non-sputum approaches for TB detection in low-resource settings. Despite these advances, hybrid AI-mechanistic pipelines remain experimental and lack standardised validation benchmarks (44, 47, 80), and are further limited by data availability and challenges in model generalisability, particularly when datasets are small or highly heterogeneous (112, 113).

In addition to these genomics and biomarker focused applications, AI and ML are also being applied to TB diagnosis using clinical and imaging data, producing real world observable signals that may help inform model calibration and validation. Orjuela Cañón et al. (114) demonstrated that supervised ML classifiers trained on real-world patient data could support TB diagnosis in routine clinical settings, including resource-limited healthcare systems. Shilpa and Ayeesha Banu (71) evaluated multiple deep-learning and hybrid models on large chest X-ray datasets, achieving high validated performance and demonstrating the strong potential of modern CXR based AI tools for reliable TB detection. Complementing these primary studies, Hansun et al. (115) synthesised evidence from many studies across multiple diagnostic modalities, showing that convolutional neural networks dominate current AI-based TB detection pipelines.

### 4.8 Parameter estimation

Credible modelling depends on appropriately selecting a model and calibrating it to existing datasets using transparent methodologies. This stage of the modelling process can be separated into model selection, model identifiability analysis, and model calibration. Model selection is the process of determining what mechanisms, rules, and behaviours are represented in the model, and is especially important when many plausible but non-determinable rules may explain extant, often low-resolution datasets. Cockrell & An (116) developed a methodology for using genetic algorithms for such problems. Much is known about the individual biological mechanisms underpinning TB outcomes (36), many of which underpin the *de facto* model selection of the foundational models reviewed in Section 3. Once a model structure is chosen (e.g., ABM rules or ODE definitions), many parameters linked to the underlying biology (often stated as numeric variables characterising the time-evolution of the model system) must be chosen.

When it is practically possible, identifiability analysis can yield two substantial benefits. First is that a unique determination of parameters means that parameter values may be fairly and directly translated as a biological result. This is because identifiability prevents parameter compensation, where errors in one parameter can be masked by adjustment of another parameter, generate equivalent model output (117). *Structural identifiability analysis*—determining whether a model could possibly be uniquely calibrated even with noiseless data (117)—can provide insights as to what future datasets would most fruitfully boost a model’s predictive power. Identifiability methodologies and software have been developed for ODE-based models (72), including those of diseases like COVID (118). However, TB models depend on sparse and messy datasets to generate heterogeneous virtual cohorts, detailed calibration, and mixed data sources, including imaging and PK/PD. Thus, *practical identifiability analysis*—determining whether a model can be uniquely calibrated given available data (117)—is not currently tractable for within-host TB models. Structural uncertainty remains an important challenge because different model structures may produce similar outputs despite relying on different biological assumptions.

While unique determination of parameter values is challenging with an immensely heterogeneous system, multiple approaches allow for estimation of parameter ranges that generate plausible simulations for downstream analysis. Model components can be structured such that parameter values are directly comparable to biological ranges measured either *in vivo* or *in vitro* (e.g., Mtb replication rates used for initial parameterisations of *HostSim* (46)). These can be improved with calibration workflows or approximate Bayesian computing with sequential Monte Carlo sampling; such workflows allow for estimation of biologically-relevant parameter ranges even with sparse and multimodal datasets (73, 81). *HostSim* was parameterised using human and non-human primate datasets and subsequently used to investigate concomitant-immunity (13, 88). There, inference of unobservable immune parameters (such as the lifespan of tissue-resident memory T cells) was demonstrated through comparison of reinfection simulations with NHP data and historical human data. Other methods like CrossLabFit have also been developed to facilitate integration of biological mechanisms into calibration via qualitative constraints (119), though to our knowledge these have not yet been applied to TB models.

## 5 Modelling TB coinfection and comorbidities

Compared to the extensive and rapidly expanding body of within-host TB infection alone models, mechanistic modelling of TB coinfection and comorbid conditions remains extremely limited (36, 37). This likely reflects the fact that coinfections and comorbidities such as HIV, diabetes, undernutrition, or viral infections such as COVID-19 can weaken immune responses, body processes, and drug behaviour in complex ways. This added complexity, together with limited within-host datasets across these conditions and variability across populations, creates significant challenges for model parameterisation, validation, and cross-scale integration. Despite their crucial clinical impact, very few studies address how HIV, diabetes mellitus (DM), or chronic obstructive pulmonary disease (COPD), including chronic bronchitis and emphysema, reshape granuloma structure, immune responses, and drug distribution. We therefore present coinfection and comorbidity focused modelling as an independent theme to highlight this major gap in literature.

Because comorbidities introduce distinct alterations to immunity and drug behaviour, we present them as separate non-mutually exclusive and non-exhaustive comorbidity themes (Sections 5.1- 5.3). These themes are: (1) TB-HIV coinfection (34, 49); (2) TB-DM (50, 78) and (3) TB-COPD and TB-Undernutrition (120). The grouping reflects differences in host-pathogen interactions associated with each condition. TB-HIV represents a coinfection characterised by direct pathogen-pathogen interactions, while TB-DM is considered separately as a systemic comorbidity with distinct metabolic and immunological effects. On the other hand, TB-COPD and TB-Undernutrition are together as host-driven comorbidities that alter host immunity and lung environment.

Historically, the development of coinfection/comorbidity models was dependent on availability of relevant biological data. For example, Kirschner’s influential 1999 HIV-TB coinfection model (34) was developed when researchers had already produced extensive HIV immunology and viral-load datasets, allowing early within-host interactions between HIV and TB to be quantified. In contrast, TB-DM modelling could not emerge until mechanistic datasets became available much more recently, such as Panda et al. (78), which experimentally measured hyperglycaemia induced macrophage dysfunction that directly informs parameterisation and immune impairment rules used in TB-DM models. Sophisticated diabetes models do exist, including “artificial pancreas” systems that simulate glucose-insulin dynamics and in silico clinical trials. However, these frameworks do not incorporate infection dynamics (121). Likewise, COPD specific inflammatory profiles required for TB-COPD models were only generated in recent years, informing the TB-COPD ABM proposed in Sershen et al. (120), which captures how COPD-driven inflammation alters TB lesion behaviour and host immunity. These studies demonstrate that coinfection and comorbidities fundamentally alter TB progression and reveal urgent gaps in our mechanistic understanding that future within-host models must address.

**Figure 4** illustrates the extreme sparsity of within-host TB coinfection and comorbidity modelling, with most *Theme ⨉ Approach* combinations represented by at most a single study. Supplementary citation-weighted analyses are presented in **Figure A2**. The observed pattern highlights a lack of methodological diversity and continuity, with current understanding constrained by a handful of isolated, high-impact studies.

For each of the coinfections we searched the literature for, there were one (or few) highly cited modelling study/studies that utilised a narrow scope of approaches. Panel A in **Figure 4** shows the modelling approaches used for coinfection and comorbidity themes. Due to the limited number of studies available, the raw and citation-weighted heatmaps are identical after normalisation (see **Figure A2A**). The row-normalised raw count heatmap (**Figure 4A**) shows this clearly for TB-COPD and undernutrition: Granuloma ABM, Hybrid ABM-ODE/PDE, and Multiscale models were each used by a single study that captures COPD-driven hypoxia and immune impairment (120). Similarly, one study of TB-DM used Omics-informed modelling, Surrogate & ML-assisted modelling approaches, while two studies used Comorbidity-informed mechanistic anchors (78, 122). As a supplementary exploratory analysis, the row-normalised citation-weighted heatmap (**Figure A2A**) indicates that scholarly impact within individual themes is often concentrated in a small number of highly cited studies rather than distributed across multiple contributing papers.

The raw count heatmaps highlight the absence of mature, cross-cutting within-host TB modelling frameworks for coinfections and comorbidities. Panel B in **Figures 4**, **A2** shows the themes that account for each modelling approach impact. In the column-normalised raw count heatmap (**Figure 4B**), the ODE/PDE models column is dominated by TB-HIV because two classical within-host ODE studies constitute almost the entire TB-HIV modelling theme. Similarly, multiscale and granuloma approaches are dominated by TB-COPD and TB-Undernutrition, each again represented by a single study. As a supplementary exploratory analysis, the column-normalised citation-weighted heatmap (**Figure A2B**) suggests a similar structure, with scholarly impact concentrated in themes where only a small number of studies exist. For example, the visible citation-weighted contribution of TB-COPD and TB-undernutrition within multiscale modelling arises primarily from a single COPD-TB multiscale study.

### 5.1 TB-HIV coinfection

HIV coinfection reshapes the immune environment needed for granuloma containment by altering cytokine signalling and reducing effector T cell availability (49). Early within-host TB-HIV models, such as Kirschner (34), analysed the systemic co-dynamics of HIV, Mtb, and immune cells using ODEs, demonstrating how HIV-driven CD4^+^ T-cell depletion and increases in viral load destabilise TB control and accelerate progression to active disease. Building on this foundation, Bauer et al. (49) developed a detailed mechanistic model of latent TB reactivation during HIV infection, incorporating macrophages, multiple subsets of T cells, and key cytokines. Their analysis indicates that HIV infection creates a markedly altered cytokine environment that weakens lung immunity, reduces the recruitment of macrophages and T cells, and ultimately drives the transition from latent to active TB. The results highlight that macrophages play a pivotal role in TB-HIV coinfection and identify them as promising targets for therapeutic intervention. Hoerter et al. (123) reviewed broader biological evidence relevant to the coinfection dynamics explored in early within-host ODE models such as those developed by Kirschner (34) and Bauer et al. (49). Although clinical evidence links ART initiation to Immune Reconstitution Inflammatory Syndrome (IRIS) risk, current within-host TB-HIV models do not explicitly simulate IRIS. Instead, they identify immunological shifts, such as rapid changes in CD4^+^ T cell availability that could contribute to IRIS-related dynamics (34). Drug-drug interactions between ART and TB regimens significantly affect granuloma penetration and efficacy, yet remain underrepresented in current within-host models (123).

### 5.2 TB-diabetes mellitus

Diabetes mellitus (DM) is a metabolic disorder characterised by high blood glucose levels that can weaken immune responses and increase the risk of infections such as TB (9). Consequently, DM is a recognised risk factor for disease progression and can influence the severity of TB and treatment outcomes by altering immune control and drug responses. TB-DM comorbidity remains one of the least modelled areas in within-host TB systems biology. To date, no mechanistic within-host mathematical models explicitly simulate TB-DM interactions. However, a growing body of immunology and multi-omics studies now defines the macrophage, cytokine, and metabolic pathways that future within-host TB-DM mathematical models will need to develop such models.

Experimental evidence shows that chronic hyperglycaemia alters macrophage activation, reduces nitric oxide (NO) and reactive oxygen species (ROS) production, and skews cytokine balance, weakening the sterilisation of granuloma (78, 122). These findings suggest that DM-driven immune dysfunction could significantly alter the dynamics of TB and the efficacy of treatment. A growing body of review literature reinforces these findings: diabetes consistently disrupts macrophage killing capacity, antigen presentation, inflammatory signalling, T cell responses, cytokine networks, lipid and metabolic pathways, and oxidative stress regulation, thereby creating immune conditions that promote TB progression (9, 124–126).

Emerging biomarker-driven machine-learning studies show that diabetic immune alterations can be used to predict TB risk. Ye et al. (50) analysed transcriptomic data from DM-TB patients and applied machine-learning methods to identify immune biomarkers that distinguish individuals at increased risk of TB. Biomarker reviews (127) highlight the metabolic, immunological, and inflammatory signatures characteristic of TB-DM comorbidity, clarifying which pathways may be the most important to integrate into future mechanistic models.

A recent review (128) also highlights autophagy as an important but currently underexplored mechanism in TB-DM comorbidity. Autophagy is a cellular defence process that helps immune cells eliminate Mtb and regulate inflammatory responses. In people with diabetes, impaired autophagic pathways may reduce bacterial clearance and potentially reducing the effectiveness of TB treatment. The review also highlighted the potential role of host-directed therapies such as metformin in enhancing autophagy-mediated mycobacterial control and improving treatment responses in TB-DM patients. This suggests that autophagy-related mechanisms should be considered in future within-host TB-DM models when investigating host-directed therapies and treatment responses. Developing within-host TB-DM models that capture these host-specific immune and metabolic impairments remains a major gap and an important priority for future TB modelling research.

### 5.3 TB and other comorbidities: COPD and undernutrition

COPD (chronic obstructive pulmonary disease), a long-term lung condition that alters airflow and lung structure, and undernutrition, a deficiency in energy and nutrient intake that weakens immune function, can reshape the granuloma microenvironment and drug distribution, increasing the risk of TB treatment failure. Within-host mathematical modelling of TB in the context of COPD and undernutrition remains underdeveloped compared to HIV and diabetes, yet emerging studies (120, 129) highlight their mechanistic importance.

Sershen et al. (120) developed a multiscale COPD-TB framework showing that COPD-related inflammation and low oxygen levels make granulomas more hypoxic, reduce the immune system’s ability to control infection, and increase the chance that Mtb spreads. Clinical immunology studies, including Lu et al. (130), show that poor nutritional status weakens immune responses and contributes to more severe pulmonary TB. A complementary narrative review by Sinha et al. (129) showed that undernutrition further impairs immune control, disrupts vaccine-induced immune responses, and increases the incidence, severity, and failure of treatment, identifying it as a major driver of susceptibility to TB and poor clinical outcomes. Undernutrition weakens host immune responses and can reduce the efficacy of the vaccine, increasing susceptibility to infection. At the population level, modelling frameworks have evaluated the epidemiological impact of nutritional interventions and other societal determinants of TB burden (131, 132). Modelling these comorbidities would allow for enhanced treatment options for many patients. Limitations remain significant: COPD and undernutrition phenotypes are not parameterised into reusable libraries; mappings from experimental data to model parameters (e.g. oxygen delivery, chemotaxis, drug diffusivity) lack standardization; and calibration against clinical endpoints is sparse (37, 47, 62, 120).

## 6 Successes, limitations, and future directions

Building on the developments in Sections 4 and 5, we focus on how the successes and limitations of within-host TB modelling will inform translational impact of TB research in the future.

### 6.1 Overall successes of within-host modelling

Within-host TB modelling has made significant advances, evolving from conceptual ODE frameworks to multiscale systems that integrate spatial heterogeneity, pharmacology, and host variability. The foundational ODE models clarified immune regulatory mechanisms and cytokine dynamics (34), while agent-based and hybrid approaches captured granuloma formation, heterogeneity, and dissemination (39, 40). A crucial advance has been the integration of PK/PD and systems pharmacology with granuloma-scale models, which revealed diffusion barriers associated with hypoxia and caseous necrosis and provided a framework for evaluating treatment regimens under realistic anatomical conditions (41–43, 80). Translational pipelines such as GEODE bridge *in vitro* drug interaction data to granuloma-level efficacy and provide a framework for prioritisation and calibration against experimental datasets (44), while virtual cohort frameworks (e.g. *HostSim*) propagate heterogeneity from granuloma to systemic scales to investigate interventions and identify host factors that may influence treatment responses (46, 64). Image calibration has allowed for more detailed simulations; PET/CT and histopathology provide structure and granuloma-level activity for parameterisation (61, 68). Transport physics models quantify oxygen/nutrient/drug gradients (62), and omics link molecular signatures to host variability (37, 45). Evolutionary and resistance modelling connects heterogeneity in drug exposure to selection pressures and heteroresistance (69, 79). Finally, AI/ML methods, surrogates, and Bayesian optimisation are leveraging patient-specific omics, imaging, and PK datasets to reduce computational burden while expanding regimen exploration within mechanistic workflows (44, 47, 80).

Alongside advances in multiscale integration and PK/PD coupling, the field is increasingly adopting systematic calibration and sensitivity pipelines, such as, CaliPro (73), that help reveal non identifiable parameters and stabilise estimation across scales. These methodologies refine ranges of parameters (or parameter combinations) that underpin multiple types of complex TB models, including ABMs, transport PDEs, and PBPK/QSP modules. Prior studies in each of these modelling domains (45–47, 61, 62, 65) highlights the high dimensionality and heterogeneity of multiscale TB frameworks and the consequent need for structured calibration approaches. Complementing this, recent methodological work on structural identifiability in ODE and age structured PDE (117) clarifies when unique inference is possible and when only identifiable combinations can be recovered, providing a rigorous mathematical foundation for interpreting multiscale TB models.

### 6.2 Limitations and future directions for within-host TB modelling

While there are great advances in modelling on Mtb infection over the past two decades, there are some clear gaps that are open areas where modelling can help make significant contributions. With regard to vaccine development and testing, most current studies exist at the epidemiological level and little work has been done at the within-host scale (133). A key paper posed the idea is the creation of a new field called “immunostimulation/immunodynamic” (IS/ID) modelling, which aims to translate mathematical frameworks used for drug dosing towards optimizing vaccine dose decision-making. Analogous to PK/PD modelling, the mathematical description of drug distribution and effects within a host, would apply mathematical models to describe underlying mechanisms by which the immune response is stimulated by vaccination (IS) and the resulting measured immune response dynamics (ID). The development of these tools could revolutionize pre-clinical vaccine development. There are a few existing within-host modelling vaccine studies (134–137) focused mainly on simulating vaccine-induced immune responses against Mtb, including cellular and cytokine-mediated control of infection and granuloma dynamics. The EU has supported the project “in **S**ilico **Tri**als for **Tu**berculosis **Va**ccine **D**evelopment” (STriTuVaD), and consortium members have calibrated an in silico pipeline to investigate a candidate therapeutic TB vaccine administered alongside antibiotics (138–141), based on the Universal Immune System Simulator (142, 143). However, prophylactic vaccine and treatment modelling remain largely disconnected, as existing treatment and regimen optimisation models rarely incorporate vaccine-induced immune states, leaving important questions regarding vaccine-treatment interactions unresolved. What becomes important is knowing which immune cells to get to the right place and when. Integrating vaccination prediction and testing into within-host TB models offers a powerful opportunity to link immune priming, granuloma-scale dynamics, and treatment response within unified frameworks. This represents an important open area and key future direction, particularly for host-variable or comorbid populations, where differences in vaccine-induced immune responses may significantly alter disease progression and treatment outcomes, both of which future models should capture explicitly aiding advancement of the field. Use of optimisation tools in this setting can be particularly powerful as well allowing narrowing of the large space of possible combinations of antigens to elicit the correct response.

As suggested, there is a dearth of models exploring co-infections. Multimorbidity has been underrepresented despite that most people who get HIV, likely have latent TB. Most current TB-HIV models still lack fully mechanistic links capturing drug-drug interactions between ART and TB medications across scales (46, 123), and COPD and undernutrition phenotypes are not yet parameterised in reusable libraries for transport–immune coupling (62, 120). Additionally, there are no immune-focused TB-diabetes models despite clear evidence of hyperglycaemia-driven macrophage and T-cell dysfunction (9, 78, 122, 124). The coinfection/comorbidity heatmaps (in Section 5) highlighted a major evidence gap: out of twenty-one *Theme ⨉ Approach* cells contained at most two studies, only seven are populated, each containing at most two studies, while the remaining fourteen are empty, underscoring how thin and uneven the TB-HIV, TB-DM, and TB-COPD and TB-Undernutrition modelling literature remains. The sparsity of these cells likely reflects the substantial biological, data, and modelling challenges inherent in coinfection and comorbidity modelling. Nevertheless, as data availability and modelling frameworks mature, these areas represent an important longer-term opportunity for expanding integrated within-host coinfection and comorbidity frameworks. Integrated TB-HIV frameworks should propagate ART-TB interactions from PBPK/QSP layers to granuloma-level PK/PD, incorporate adherence variability, and quantify immune reconstitution inflammatory syndrome (IRIS) alongside sterilisation probabilities calibrated against imaging-anchored granuloma heterogeneity (46, 62, 123). Immune-focused TB-DM models should encode hyperglycaemia-induced deficits in macrophage activation, NO/ROS production, and cytokine thresholds, together with diabetes-related PK changes in clearance and binding; model validation should ensure that predictions are consistent with both experimental immune function measurements and clinical outcomes, such as the time required for sputum cultures to convert to negative (78, 122, 124). Reusable phenotype libraries for COPD and undernutrition, parameterising oxygen delivery, matrix remodelling, chemotaxis, effector recruitment, and drug diffusivity could facilitate scenario testing of granuloma-targeted strategies, including inhaled or locally intensified regimens (62, 66, 120). To strengthen calibration and comparability, the community should release open, paired datasets that combine PET/CT, histology, microenvironment metrics, and PK profiles with parameter dictionaries and unit conventions, alongside benchmark tasks (e.g. reproducing transport gradients, ranking BPaL variants across standardised heterogeneity panels) for fair model-to-model evaluation (44, 61, 62, 64). The lack of mechanistic models limits the ability to accurately predict disease progression or design interventions tailored to co-affected people, highlighting the need for future work to develop dedicated coinfection/comorbidity-focused models.

Several important immunological processes, including macrophage heterogeneity, neutrophil biology, tissue-resident immunity, immunometabolism, immune exhaustion, granuloma vascularisation, and host genetic diversity, are not yet fully captured in current within-host TB models (37, 46, 62). Their inclusion is often limited by biological uncertainty, scarce data, and the additional complexity they introduce. Improved representation of these processes may strengthen understanding of disease progression, host heterogeneity, and treatment response.

Coinfection notwithstanding, detailed within-host simulations are limited due to the complexity and computational cost of linking multiple modelling modules, each representing TB processes across different spatiotemporal scales. Two-way coupling between granuloma-scale ABMs/PDEs, systemic immune compartments, and PBPK/QSP pharmacology remains technically demanding, with numerical stability and computational costs (46, 47). Overcoming this will involve efficiently linking scales, requiring streamlined techniques that allow modellers to connect extant granuloma-scale ABMs, transport PDEs, systemic immunity, and PBPK/QSP pharmacology through clear and consistent interfaces for shared variables, units, and time-steps, supported by standardised code examples and schematic diagrams (44, 46, 47). Some frameworks exist to orchestrate integration of multiple simulators, though without incentive initiatives the upfront costs will likely slow the development and adoption of such platforms (144).

Human granuloma-specific PK/PD data are sparse, and mappings from imaging/histology to model parameters (e.g. diffusivity, necrosis fraction, immune cell densities) lack standardisation, complicating predictions of granuloma sterilisation and comparisons across models (61, 62, 64). Even within single-disease contexts, regimen rankings can be sensitive to spatial resolution, detection limits, and initial bacterial burden (64). Harmonising metrics for antibiotic regimen efficacy (e.g., what do we mean by “cured” TB in simulation)? is challenging without a *lingua franca* or a repository for how we relate model state to disease state.

A major underlying constraint across multiscale TB models is parameter identifiability. In high dimensional systems that include granuloma level transport processes, immune activation thresholds, and drug-drug interaction terms, many parameters are only weakly or indirectly informed by available data, and those datasets. Further, TB outcomes are based on many factors that we do not represent (or necessarily fully know), and can be impacted by dozens of extrinsic factors such as undernutrition, age, and genetics, leading to considerable within-host and between-host heterogeneity (5). Identifiability methods are not standardised for ABMs or PDEs, thus any *ad hoc* methods for identifiability would need to be re-visited whenever the model is restructured for the inclusion of new biology or when representing comorbidities. Lacking this, parameter compensation can lead to divergent mechanistic interpretations or regimen rankings despite similar goodness of fit (37, 46, 47, 62, 65).

As TB models continue to expand in dimensionality, integrating omics, PK/PD granuloma scale, and computationally intensive virtual patient cohorts, it is unlikely that any sufficiently-detailed, mechanistic, personalised model of TB infection will be identifiable under the current paradigm (37, 47). A first-time model that translated omics data directly into a mathematical model was done creating a four-step analysis approach for comprising: filling missing data and harmonizing data sources, inducing sparsity, developing mechanistic models, and interpretation (145) This approach has not yet been applied for diseases and could revolutionize the translation of omics data directly to modelling. Detailed within-host models of TB must reconcile small and heterogeneous datasets from disparate sources; to simulate drug-susceptible antibiotic treatment *HostSim* parameterises over 100 parameters by reconciling human-equivalent PK data from rabbits, population-distribution of infection outcomes from humans, transcriptomic data for drug-drug interactions, and microbiological/immunological data from macaques (64). A model capable of capturing the heterogeneity of TB treatment for personalised medicine will need to include additional features such as age, genetics, and potentially metabolomic data to capture nuances of treatment. More flexible frameworks for modular identifiability will likely be required; wherein model components may be calibrated and identified in isolation before being coupled into a larger model. Such an approach would allow us to leverage known (possibly qualitative) biological mechanisms as-studied-in-isolation as a form of “data” for identifiability. This has precedent; recent identifiability study of virus infection models have shown that identifiability is improved when multi-component models are analysed together (146); this indicates that careful coupling of multi-component models can improves the identifiability of the overall system.

Reproducibility remains uneven: parameter provenance, calibration datasets, sensitivity/uncertainty methods, and code/data availability are inconsistently reported, limiting model-to-model comparison and credibility assessment (37, 47, 147). Recent efforts have advocated for the findable, accessible, interoperable, and reproducible (FAIR) principles of data stewardship (148) to be adopted by computational modellers containerised environments. Reviews consistently emphasise reproducibility as the cornerstone of credibility, advocating transparent parameterisation and harmonised data standards (37, 47). Reproducibility will depend on transparent calibration pipelines, open sharing of detailed methodologies and datasets, and harmonised reporting practices. Future studies should consistently include full descriptions for any model parameterisation, uncertainty quantification, sensitivity analysis, and identifiability metrics to clearly communicate which inferences are well supported by the data. Beyond parameter uncertainty, structural uncertainty remains an important challenge and highlights the need for further model comparison and validation.

A significant step in developing our understanding of within-host TB dynamics lies as much in our ability to characterise its scope—what the model does or does not claim, what it *may* claim, and what problems it can credibly address. The heatmaps in **Figures 3**, **A1** show that established models are used to address a wide array of topics, whereas emerging technologies have focused on comparatively narrow themes. Who is to say *a priori* how important is it that a model includes, say, AI components or is identifiable? Do PDE and ABM models need to include genetics if their conclusions speak directly to genetic impacts, or does a coarse-grained ODE model suffice? In some cases, the answer is straightforward. A model must be mechanistic when its results claim to elucidate mechanisms about a complex system (149). If a model is identifiable, then parameter values are translatable and can be considered as a credible result of that model; thus, if the model reports the parameters as a key result, the model must be identifiable. Is the same for predicting non-identifiable ranges? Appropriate assessment of scope is also critical for determining whether a personalised model is “personalised enough” for a given use-case.

The veritable zoo of models used to study TB have distinct foci, simplifications and assumptions based on the questions that each model speaks to. Many of these simplifications and assumptions may impact model scope and may not be reported rigorously or completely as they could be but are typically harmless due to the peer review process. As models get larger, and as our questions become more detailed, we as modelers risk becoming siloed with non-rectifiable models, as reflected in **Figure 3**, unless we all communicate precisely what biology our models do and do not represent. In future, each TB model should include a map that details not only what is represented, but what aspects of that representation are *claiming to reliably reflect reality* - a map of scope. This is distinct from FAIR guidelines (which focus on the critically important reproducible *implementation*), but we call for a map of *translatability claims*. Consistently defining maps in a human-readable, claim-based synthesis will keep the relevant biological justifications for a model portable. On the horizon, generating and compiling these maps into a communal atlas of TB models by-scope could allow modellers to rapidly determine what extant models could be assembled to create complex, detailed simulators appropriate to approach specific questions.

Recent extensions of within-host modelling frameworks to non-tuberculous mycobacterial infections highlight the broader applicability of the eight pillars outlined in this review. Notably, agent-based and multiscale models capture host immune dynamics, spatial heterogeneity, and bacterial behaviour within lung tissue (150). These approaches build on foundational multiscale frameworks originally developed for TB granuloma formation (42), demonstrating how core modelling principles can be extended to related mycobacterial diseases.

## 7 Conclusion

Tuberculosis is a complex disease that has been with humans for millennia. Experimental and clinical studies have not yet found lasting solutions, and mathematical and computational modelling can assist with testing of ideas, predicting mechanisms and exploring treatment and vaccine design spaces.

Within-host tuberculosis modelling has evolved from conceptual ODE frameworks to sophisticated multiscale systems that integrate spatial heterogeneity, pharmacology, and host variability. These advances have transformed modelling from a theoretical exercise into a translational tool capable of informing regimen design and virtual clinical trials. Agent-based and hybrid models have further highlighted how granuloma architecture, hypoxia, and caseous necrosis influence drug penetration and treatment outcomes (41–43, 60, 62). Imaging and omics have further strengthened the credibility of the model, enabling calibration against granuloma-level structure and molecular signatures (45, 61, 68). Virtual cohorts now propagate variability across scales, while AI/ML approaches accelerate optimisation and uncertainty quantification (44, 46, 47, 80).

Despite these successes, critical gaps remain. Multiscale models are parameter-rich, structurally complex, and often constrained by limited or noisy data. Granuloma-specific pharmacology under TB-HIV coinfection is underrepresented, with ART-TB drug-drug interactions rarely propagating from the systemic PBPK/QSP layers to granuloma PK/PD (46, 123). Immune-focused TB-diabetes models do not yet exist, and COPD and undernutrition phenotypes lack parameterised libraries (78, 120, 122, 124). Calibration, identifiability, and reproducibility challenges persist, with inconsistent reporting of parameter provenance and calibration datasets (37, 47, 147). Addressing these limitations will require modular architectures, open calibration bundles, and harmonised outcome metrics. The vast variety of TB models, often constructed to remedy a scarcity of relevant data, makes it difficult to systematically determine what models are appropriate for what tasks and contexts, which in turn makes it even more challenging to determine the credible scope of larger, coupled, multiscale models with sufficient detail to capture the true heterogeneity of TB.

Future advances in within-host TB modelling will require unified frameworks that integrate vaccination, infection dynamics, and treatment response to improve predictive accuracy and translational impact. Extending these approaches to related infections, such as non-tuberculous mycobacterial disease, represents an important step towards a broader understanding of mycobacterial pathogenesis and treatment.
