# Supplementary material for: The eight pillars of within-host tuberculosis modelling
Source: Front Immunol. 2026 Sep 3;17:1920524. doi: 10.3389/fimmu.2026.1920524 (PMC13584441; doi:10.3389/fimmu.2026.1920524)
Supplement: Supplementary file 1 [file DataSheet1.pdf]

Supplementary Materials: Heatmap data and python code for The Eight Pillars of Within-host Tuberculosis Modelling manuscript

Overview
These supplementary materials contain the data and code used to generate the heatmaps presented in the manuscript. The heatmaps illustrate the distribution of modelling approaches across key thematic areas in TB within-host modelling, including TB comorbidities.

Files included

1. ***TB_heatmaps_data.xlsx***
   Excel workbook containing the data used to generate the heatmaps. It includes the following worksheets:
   - Sec4_raw_counts
   - Sec5_raw_counts
   - Sec4_weighted_counts
   - Sec5_weighted_counts
2. ***make_tb_heatmaps.py***
   Python script used to load the data, process it, and generate the heatmaps.

Requirements
The script was developed in Python and requires the following packages:

- numpy
- pandas
- matplotlib
- seaborn
- openpyxl

Install dependencies using:
pip install numpy pandas matplotlib seaborn openpyxl

How to run
Ensure that both the Python script and the Excel file are located in the same folder. Then run:
python make_tb_heatmaps.py --input TB_heatmaps_data.xlsx

What the Script Does
The script:

1. Loads data from the Excel file using the `load_sheet` function.
2. Processes raw and weighted counts from four worksheets in the Excel file (Sec4_raw_counts, Sec5_raw_counts, Sec4_weighted_counts, and Sec5_weighted_counts), preparing them for analysis and visualisation.
3. Applies row- and column-wise normalisation using the `row_norm` and `col_norm` functions .
4. Generates heatmaps using the `plot_heatmap` function.
5. Saves the resulting figures to file.

Data Description

- *Rows* represent thematic categories (e.g. *Multiscale & Multicompartment Representation*, *TB-HIV*).
- *Columns* represent modelling approaches (e.g. ODE/PDE models, ABM, ML-assisted models)
- Values correspond to:
  -Raw counts
  -Citation-weighted counts

Reproducibility
All heatmaps included in the manuscript can be reproduced directly from the provided data and script by running the command above.
