# Supplementary material for: The eight pillars of within-host tuberculosis modelling
Source: Front Immunol. 2026 Sep 3;17:1920524. doi: 10.3389/fimmu.2026.1920524 (PMC13584441; doi:10.3389/fimmu.2026.1920524)
Supplement: Supplementary file 2 [file DataSheet2.pdf]

```
#!/usr/bin/env python3
"""
make_tb_heatmaps.py (UPDATED)
Supports:
 - Sec4_raw_counts
 - Sec5_raw_counts
 - Sec4_weighted_counts
 - Sec5_weighted_counts
Generates raw and weighted heatmaps for Section 4 and Section 5.
"""

import argparse
from pathlib import Path
import numpy as np
import pandas as pd
import seaborn as sns
import matplotlib.pyplot as plt


# ============================================================
# FIXED THEME ORDER
# ============================================================

SEC4_ORDER = [
    "Omics & Host Variability",
    "Virtual Clinical Trials & Regimen Optimisation",
    "Multiscale & Multicompartment Representation",
    "Translational Impact & Precision Medicine",
    "Imaging-informed Studies",
    "Evolutionary Dynamics & Resistance",
    "Emerging AI / Machine Learning ",
    "Parameter Estimation",
]

SEC5_ORDER = [
    "TB-HIV",
    "TB-DM",
    "TB-COPD & TB-Undernutrition",
]


# ============================================================
# DATA LOADING
# ============================================================

def load_sheet(xlsx: Path, name: str) -> pd.DataFrame:
    """
    Load a worksheet and convert it to float DataFrame.
    """
    df = pd.read_excel(xlsx, sheet_name=name, engine="openpyxl")
    df = df.set_index(df.columns[0])
    df = df.apply(pd.to_numeric, errors="coerce").fillna(0.0)
    return df.astype(float)


def reorder(df: pd.DataFrame, desired_order: list[str]) -> pd.DataFrame:
    missing = [t for t in desired_order if t not in df.index]
    if missing:
        raise ValueError(f"Missing themes in sheet:\n" + "\n".join(missing))
    return df.loc[desired_order]


# ============================================================
```

```
# NORMALISATION
# ==============================================================

def row_norm(df: pd.DataFrame) -> pd.DataFrame:
    rowsum = df.sum(axis=1).to_numpy()
    mat = df.to_numpy()
    out = np.divide(mat, rowsum[:, None],
                    out=np.zeros_like(mat),
                    where=rowsum[:, None] != 0)
    return pd.DataFrame(out, index=df.index, columns=df.columns)


def col_norm(df: pd.DataFrame) -> pd.DataFrame:
    colsum = df.sum(axis=0).to_numpy()
    mat = df.to_numpy()
    out = np.divide(mat, colsum[None, :],
                    out=np.zeros_like(mat),
                    where=colsum[None, :] != 0)
    return pd.DataFrame(out, index=df.index, columns=df.columns)


# ==============================================================
# PLOTTING
# ==============================================================

def plot_heatmap(df, title, outfile, cmap="YlGnBu"):

    fig_h = max(6, 0.45 * len(df))
    fig_w = 12 if "Comorbidities" in title else 10  # wider for Section 5
    fig, ax = plt.subplots(figsize=(fig_w, fig_h))

    sns.heatmap(
        df, ax=ax,
        cmap=cmap, vmin=0.0, vmax=1.0,
        linewidths=0.5, linecolor="white",
        cbar=True, annot=False,
    )

    ax.set_title(title, fontsize=18, fontweight="bold")
    ax.set_xlabel("Approach", fontsize=18)
    ax.set_ylabel("Theme", fontsize=18)




    # Normal rotation for Section 4
    plt.xticks(rotation=45, ha="right", fontsize=14)
    plt.yticks(fontsize=14)


    # Force horizontal theme labels for Section 5 only
    if "Comorbidities" in title or "TB Coinfections" in title:
        ax.set_yticklabels(ax.get_yticklabels(), rotation=0, ha="right")
        plt.subplots_adjust(left=0.30)
    else:
        ax.set_yticklabels(ax.get_yticklabels(), rotation=0)

    plt.tight_layout()
    fig.savefig(outfile, dpi=300, bbox_inches="tight")
    plt.close()
    print(f"[OK] Saved {outfile}")
```

```
# ================================================================
# MAIN
# ================================================================

def main():
    parser = argparse.ArgumentParser()
    parser.add_argument("--input", required=True)
    args = parser.parse_args()

    xlsx = Path(args.input)

    # ------------------------
    # READ ALL 4 NEW SHEETS
    # ------------------------
    sec4_raw = reorder(load_sheet(xlsx, "Sec4_raw_counts"), SEC4_ORDER)
    sec5_raw = reorder(load_sheet(xlsx, "Sec5_raw_counts"), SEC5_ORDER)

    sec4_wt = reorder(load_sheet(xlsx, "Sec4_weighted_counts"), SEC4_ORDER)
    sec5_wt = reorder(load_sheet(xlsx, "Sec5_weighted_counts"), SEC5_ORDER)

    # ------------------------
    # PLOT SECTION 4  RAW
    # ------------------------
    plot_heatmap(row_norm(sec4_raw),
            "Advances in TB Modelling\n (row-normalised counts)",
            "section4_raw_row.png")

    plot_heatmap(col_norm(sec4_raw),
            "Advances in TB Modelling\n (column-normalised counts)",
            "section4_raw_col.png")

    # ------------------------
    # PLOT SECTION 4  WEIGHTED
    # ------------------------
    plot_heatmap(row_norm(sec4_wt),
            "Advances in TB Modelling\n (citation-weighted, row-normalised)",
            "section4_weighted_row.png")

    plot_heatmap(col_norm(sec4_wt),
            "Advances in TB Modelling\n (citation-weighted, column-normalised)",
            "section4_weighted_col.png")

    # ------------------------
    # PLOT SECTION 5  RAW
    # ------------------------
    plot_heatmap(row_norm(sec5_raw),
         "TB Coinfections & Comorbidities\n (row-normalised counts)",
         "section5_raw_row.png",
         cmap="YlOrRd")

    plot_heatmap(col_norm(sec5_raw),
         "TB Coinfections & Comorbidities\n (column-normalised counts)",
         "section5_raw_col.png",
         cmap="YlOrRd")

    # ------------------------
    # PLOT SECTION 5  WEIGHTED
    # ------------------------
    plot_heatmap(row_norm(sec5_wt),
         "TB Coinfections & Comorbidities\n (citation-weighted, row-normalised)",
         "section5_weighted_row.png",
         cmap="YlOrRd")
```

```
    plot_heatmap(col_norm(sec5_wt),
            "TB Coinfections & Comorbidities\n (citation-weighted, column-normalised)",
            "section5_weighted_col.png",
            cmap="YlOrRd")


if __name__ == "__main__":
    main()
```
