# Supplementary material for: The eight pillars of within-host tuberculosis modelling
Source: Front Immunol. 2026 Sep 3;17:1920524. doi: 10.3389/fimmu.2026.1920524 (PMC13584441; doi:10.3389/fimmu.2026.1920524)
Supplement: Supplementary file 3 [file SupplementaryFile1.pdf]

Appendix: The Eight Pillars of Within-host Tuberculosis Modelling

Appendix A

A1 Theme ⨉ Approach Matrix

Tables A1-A4 provide the mappings connecting references to themes and approaches, as well as our calculated raw and citation-weighted representation for the analysis we describe in Methods.

Table A1: *Theme ⨉ Approach* matrix used for analyses in Section 4. Each row/column entry shows citation indices of papers published during/after 2017 using a specific approach and involving a specific theme. Headers shortened with numeric keys: (1) ODE/PDE models; (2) hybrid ABM-ODE/PDE models; (3) multiscale models; (4) PBPK/QSP framework; (5) granuloma-based ABM; (6) whole-lung/metapopulation models; (7) virtual cohorts/virtual clinical trial; (8) omics-informed modelling (9) calibration/ UQ; (10) surrogate and ML-assisted modelling.

**Theme**	**(1)**	**(2)**	**(3)**	**(4)**	**(5)**	**(6)**	**(7)**	**(8)**	**(9)**	**(10)**
Omics & Host Variability	(45)		(45)	(45)	(45)			(63)		(45)
Virtual Clinical Trials & Regimen Optimisation		(65)	(46,64, 65)	(44,65, 74,80)	(46,64, 65)		(46,64,65)			(44,74,80)
Multiscale & Multicompartment Extensions	(45, 62)	(65,67,89)	(13,45, 46,65, 66,75,84,88)	(45,65)	(45,46, 65,75, 89)	(66, 75)	(46,65)			(45)
Translational Impact & Precision Medicine	(45, 62)	(65)	(45,46, 65,84)	(45,65)	(45,46,65)		(46,65)	(96)		(44,45,74, 77,80)
Imaging-informed Studies	(62)		(61)			(97–99)			(84)	(71)
Evolutionary Dynamics & Resistance								(69,70,76,100)		
Emerging AI/machine-learning				(44,80)						(30,44,45, 71,74,77, 102,104,105,107–109,114)
Parameter Estimation			(13,88)						(73,117,119)	

Table A2: *Theme ⨉ Approach* matrix for analyses used in Section 5. Entries show citation indices. Headers shortened with numeric keys: (1) ODE/PDE models; (2) hybrid ABM-ODE/PDE models; (3) multiscale models; (4) granuloma-based ABM; (5) omics-informed modelling; (6) surrogate and ML-assisted modelling; (7) comorbidity-informed modelling.

Theme	(1)	(2)	(3)	(4)	(5)	(6)	(7)
TB-HIV	(34,49)						
TB-diabetes Mellitus					(50)	(50)	(78,122)
TB COPD and undernutrition		(120)	(120)	(120)			

Table A3: Summary of Section 4 References with Citation Weights. Citation counts are as of June 2026, searched via Google Scholar. Weight is calculated as ($w_i = c_i/c_{\max}$) where c_{max} = 185.

Short label	Citation count	Weight
Martin (2017)(98)	185	1.000
White (2017)(97)	115	0.622
Larkins-Ford (2021)(30)	88	0.476
Sawyer (2023)(61)	83	0.449
Ma (2019)(102)	63	0.341
Castro (2021)(79)	61	0.330
Larkins-Ford (2022)(104)	55	0.297
Orjuela (2022)(114)	47	0.254
Joslyn (2022)(88) *HostSim*	45	0.243
Renardy (2022)(117)	41	0.222
Bowness (2018)(67)	38	0.205
Wessler (2020)(75)	37	0.200
Joslyn (2020)(73)	34	0.184
Zheng (2024)(77)	32	0.173
Cicchese (2021)(45)	28	0.151
Budak (2023)(74) Optimizing	24	0.130
Fonseca (2025)(96)	19	0.103
Samabarey (2024)(105)	18	0.097
Morales-Arce (2021)(69)	18	0.097
Marino (2018)(89)	16	0.086
Pitcher (2020)(66)	14	0.076
Michael (2023)(46)	12	0.065
Budak (2024)(65) Systematic	11	0.059
Joslyn (2022)(13) Concomitant	11	0.059
Worakitchanon (2024)(100)	10	0.054
Datta (2024)(62)	9	0.049
Shilpa (2025)(71)	8	0.043
Eams (2023)(108)	6	0.032
Ismail (2025)(76)	4	0.022
Krupinsky (2025)(84)	4	0.022
Maiello (2025)(99)	3	0.016
Michael (2025)(64)	2	0.011
Blanco (2025)(119)	2	0.011
Budak (2025)(44) GEODE	1	0.005
Cox (2025)(63)	1	0.005
Budak (2025)(80) Novel	0	0.000
Xu (2025)(70)	0	0.000
Johnson (2025)(107)	0	0.000
Li (2026)(109)	0	0.000

Table A4: Summary of Section 5 References with Citation Weights. Citation counts are as of June 2026, searched via Google Scholar. Weight is calculated as ($w_i = c_i/c_{\max}$) where c_{max} = 171.

Short label	Citation count	Weight
Kirschner (1999)(34)	171	1.000
Bauer (2008)(49)	42	0.246
Panda (2022)(78)	23	0.135
Sershen (2023)(120)	19	0.111
Chaubey (2024)(122)	10	0.058
Ye (2025)(50)	8	0.047

A2 Example computations of raw and citation-weights

The Methods Sections 2.3-2.4 describe how we compute raw and citation-weighted heatmaps. Table A5 shows illustrative examples of how we calculate both types of weights. Figures A1 and A2 show the heatmaps based on those examples.

Table A5: Example calculation of citation weights.

**Section**	**Original indices in *Theme* ✕ *Approach* cell**	**Cell total (raw)**	**Citation counts** c_i	c_{max} **per section**	**Citation-weighted cell total**
4	Cicchesse (2021)(45); Datta (2024)(62)	2	30; 9	185 (See Table A3)	(30 + 9)*/185* = 0.211
5	Sershen (2023)(120)	1	19	171 (See Table A4)	19/171 = 0.111

A3 Supplementary figures

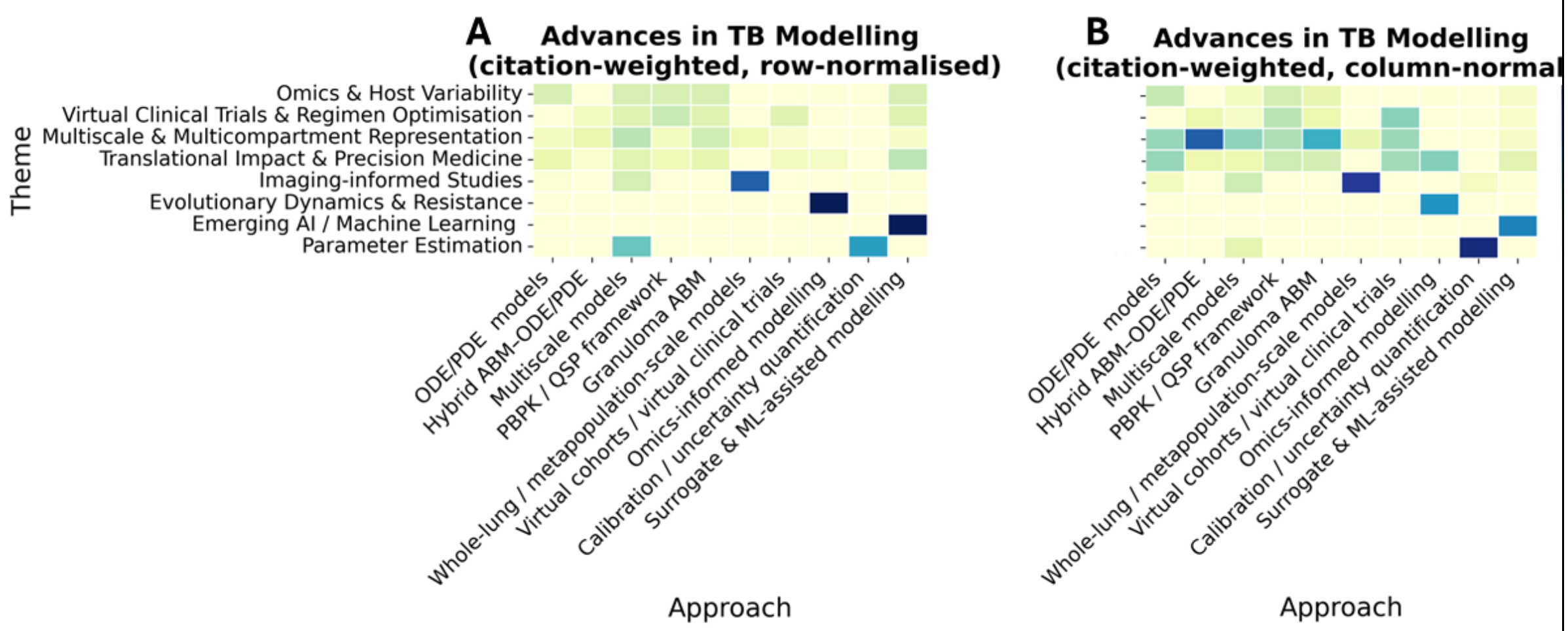

Figure A1: Research impact of post-2017 modelling studies by theme and approach. Heatmaps normalised by row (A) and column (B) of modelling approaches across post-2017 TB research themes (Section 4), based on citation-weighted counts of selected studies. The x-axis represents modelling approaches, and the y-axis represents post-2017 research themes. In panel (A), each row is normalised to sum to 1, showing the proportion of each modelling approach within a given research theme. In panel (B), each column is normalised to sum to 1, illustrating how each research theme contributes to a given modelling approach. The intensity of the colour (as indicated by the colour bar) represents the normalised proportion of citation-weighted counts (range 0-1), with lighter colours indicating lower citation-weighted impact and darker colours indicating higher citation-weighted impact. These heatmaps reflect the relative influence of studies based on citation weighting, in contrast to Figure 3, which reflects the coverage of the research based on the number of selected studies (raw counts).

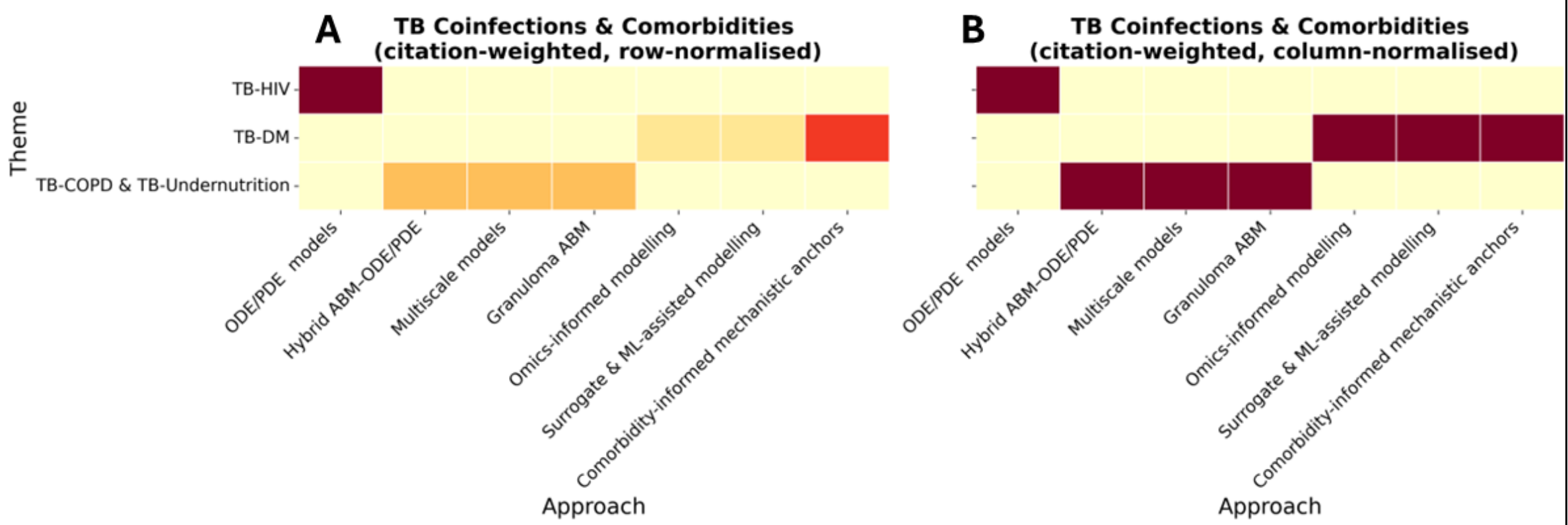

Figure A2: Research impact of TB modelling studies across coinfections and comorbidities by modelling approach. Heatmaps normalised by row (A) and column (B) of modelling approaches across TB coinfections and comorbidities (Section 5), based on citation-weighted counts of selected studies. The x-axis represents the modelling approaches, and the y-axis represents the TB coinfection and comorbidity themes. In panel (A), each row is normalised to sum to 1, showing the proportion of each modelling approach within a given comorbidity theme. In panel (B), each column is normalised to sum to 1, illustrating how each coinfection and comorbidity theme contributes to a given modelling approach. The intensity of the colour (as indicated by the colour bar) represents the normalised proportion of citation-weighted counts (range 0-1), with lighter colours indicating lower citation-weighted impact and darker colours indicating higher citation-weighted impact. These heatmaps reflect the relative influence of studies based on citation weighting, in contrast to Figure 4, which reflects the coverage of the research based on the number of selected studies (raw counts).
